# An Investigation of the Spatial Arrangement of Mycotoxin Build-Up in Corn Stored Under Different Environmental Conditions

**DOI:** 10.3390/toxins16120508

**Published:** 2024-11-25

**Authors:** Ruth Kerry, Ben Ingram, Hamed K. Abbas, Gene Ahlborn

**Affiliations:** 1 Department of Geography, Brigham Young University, Provo, UT 84602, USA; 2 Facultad de Ingeniería, Universidad de Talca, Camino a Los Niches Km. 1, Curicó 3344158, Chile; 3 USDA-ARS, Stoneville, MS 38776, USA; 4 Department of Nutrition, Dietetics & Food Science, Brigham Young University, Provo, UT 84602, USA

**Keywords:** spatial variation, mycotoxins, aflatoxin, deoxynivalenol, fumonisin, zearalenone

## Abstract

Mycotoxins are toxins produced by fungi that contaminate many key food crops as they grow in the field and during storage. Specific mycotoxins are produced by different fungi. Each type of fungus and mycotoxin have their own optimal temperatures and water activities for growth and production. The legislative limits for various mycotoxins in foodstuffs to protect human health vary between countries but all commodities have their levels evaluated based on the concentrations from one aggregated grain sample. This approach assumes that the variation in toxin levels is uniform and random without spatial trends. This study investigates the spatial distribution of four mycotoxins (aflatoxin, deoxynivalenol, fumonisin and zearalenone) in bins of clean and dirty corn when stored in an environmental cabinet for two months under different temperature and humidity conditions. The bins of clean and dirty corn each had 12 CO_2_/humidity/temperature sensors installed in three layers, and samples were extracted for mycotoxin analysis from locations close to each sensor following storage. Using Mann–Whitney U and Kruskal–Wallis H statistical tests, significant differences were found between mycotoxin levels attributable to the different environmental conditions and spatial locations of samples. Variations in aflatoxin and zearalenone concentrations were most pronounced for the range of temperature and humidity conditions chosen. By understanding the patterns of spatial variability in mycotoxin concentrations and identifying zones at high risk of contamination, as well as what conditions are favorable, targeted interventions could be implemented to reduce food waste. This work also has implications for how levels of mycotoxins in foodstuffs are sampled and measured.

## 1. Introduction

Mycotoxins are a group of toxic substances produced by fungi [1]. Fungi and their mycotoxins can contaminate staple foodstuffs when they develop on crops in the field or in grains stored for distribution [2,3]. The critical levels of several mycotoxins allowed in food and animal feeds are legislated both nationally and internationally but vary by toxin, foodstuff and between countries [4]. Some of the strictest legislative limits for mycotoxins are found in Europe [5]. Legislation is necessary due to the known adverse health effects of these toxins in humans and animals [1,6]. Aflatoxins are probably the mycotoxin of greatest concern to human health because they are category 1A known human carcinogens, and they have an established causal relationship with liver cancer [6,7]. Indeed, aflatoxin B_1_ is the most potent known hepatocarcinogen [8]. The discovery of the severe carcinogenic properties of aflatoxins in the 1960s and the adverse health effects of other mycotoxins [1] led to the start of regulation for aflatoxins and mycotoxins in general [9,10]. However, regulation does not exist in all countries and is only applied to products for export in many African countries [10].

Due to the lack of aflatoxin legislation, some less developed countries have 2–10 times [11] or 16–31 times [12] higher rates of liver cancer mortality than more developed countries. It has been suggested that the higher rates of liver cancer mortality in less developed countries are associated with aflatoxin contamination. Indeed, it has been estimated that aflatoxin exposure through consumption of contaminated food may be responsible for 4.6–28.2% of global liver cancer [12,13]. As much as 20% of food products worldwide are substantially contaminated with aflatoxins [14] and 60–80% of food crops contain detectable levels of mycotoxins [15].

The optimal conditions for many fungi to grow and produce mycotoxins have been studied in detail in laboratory settings [16,17,18,19]. The optimal conditions for fungal growth and mycotoxin production associated with four mycotoxins that are often measured together have been summarized in Table 1. While the human and animal health effects are considered most severe for aflatoxin, which is the main focus of this study, single mycotoxins rarely occur in isolation and detrimental effects have been observed with each of these toxins. Deoxynivalenol has been associated with nausea, vomiting, lack of weight gain and reproductive problems in humans and animals [20]. Fumonisin has been associated with esophagus, kidney or liver cancer and fetal death in various animals and humans [21]. Zearalenone has estrogenic effects which have been associated with various fertility issues in many animals and it has also been associated with liver cancer [22].

The primary conditions that must be maintained for optimal fungal growth and mycotoxin production are temperature and water activity expressed as equilibrium relative humidity (ERH). ERH describes the amount of water in the air surrounding the grain when in equilibrium with water in the grain. If air in contact with the grain has a relative humidity (RH) lower than the equilibrium value, water is removed from the grain by the air, which reduces the grain’s moisture content and increases the RH of the air [23]. The reverse pattern can also occur. The complexity of measuring ERH of a storage environment makes it difficult to establish links between ERH and the RH of where crops are grown and stored. Thus, it is difficult to extrapolate the meaning of all the work that has been conducted in labs (Table 1) to field and storage settings. However, there are significantly higher rates of liver cancer related to aflatoxin contamination in countries with hot moist climates compared to countries with hot dry climates [10]. Similarly, aflatoxin levels in the USA are greatest in the south-east where temperatures and RH levels are high [24].

Associated with the difficulties of relating ERH in the lab to RH in the environment, there has been very little investigation of spatial variation in toxin build-up within fields and within stored grain. A 30-year survey of aflatoxin levels in corn from Southern Georgia, USA, has been used to investigate the environmental conditions favoring aflatoxin build-up in corn in the field and found the highest risk of contamination in years and areas experiencing high temperatures and drought in June at the delicate mid-silk growth stage [25,26]. Drought can increase aflatoxin contamination because as the fungi become stressed, they produce more aflatoxins [27]. Plant stress during drought can also contribute to aflatoxin production as the plant stress hormone Methyl Jasmonate can increase the production of aflatoxin by *Aspergillus parasiticus* [28], yet it decreases aflatoxin production by *Aspergillus flavus* [29,30]. High-risk zones for aflatoxin contamination within individual fields have been associated with sandy soils and shedding topographic positions that are more drought-prone [31]. Such drought stress has been identified with the normalized difference vegetation index (NDVI) and thermal infra-red data from remotely sensed imagery [31,32]. This type of spatial analysis can help to identify zones at different levels of risk of contamination. Once identified, mitigation strategies can be implemented in high-risk zones such as targeted application of fungicides, planting earlier, irrigating and using resistant varieties. Harvesting and storing grain from different risk zones separately as micro-batches can also prevent uncontaminated grain being wasted.

Due to inter-year variability in climate and uncertainty over the timing of climate change, there is a great deal of uncertainty over where and when to apply mitigation strategies to reduce mycotoxin contamination risk [33,34,35]. Several studies have shown that aflatoxin contamination is likely to increase globally and expand its extent to higher latitudes as temperatures rise [27,36,37,38,39,40,41,42,43].

Since mycotoxin legislation was introduced, there have been substantial economic losses for farmers in regions prone to contamination. Aflatoxin leads to substantial economic losses due to yield loss, reduced crop value, a loss of animal productivity and the cost of monitoring and mitigation. These losses have been estimated to be up to USD 250 million annually in the USA for corn [44]. The loss of grain to aflatoxin contamination in the USA is tracked at the county level by the USDA Risk Management Agency (RMA). Based on the predictions mentioned above relating to increased aflatoxin contamination with climate change, it is clear that these losses are likely to increase in the near future. Aflatoxin-related crop losses need to be reduced to safely produce the 56% more food needed [45] to feed a world population of 9 billion by the year 2050 [46].

The separate storage of grain from different risk zones within growing regions and individual fields could be a significant way to reduce waste of uncontaminated grain. However, more needs to be known about the spatial variation in mycotoxin build-up in storage settings. As Table 1 shows, much mycotoxin research has concentrated on determining the optimal temperature and ERH conditions for fungal growth and toxin production in different grain products under laboratory conditions. Aflatoxin, for example, develops in hot humid storage conditions, but spatial analysis of stored grain commodities has been minimal. Given the expense of aflatoxin testing (around USD 150 per sample in commercial labs), the standard sampling protocols involve collecting several samples and mixing them into one composite sample for analysis [47,48]. This approach ignores spatial variation, so very few spatial studies have been completed for stored grain. Kerry et al. (2021) [49] simulated proportions of wasted grain given the policy of testing just one composite sample from grain piles and ignoring spatial variation. When there is a highly skewed distribution of a toxin in grain, as much as 74% can have safe concentrations when the average concentration exceeds FDA limits (see Table 1). The average concentration exceeding FDA limits means that a whole batch of grain could be discarded or sold for a more restricted purpose, lowering farmer income. In the USA, the FDA has two limits of 20 ppb and 100 ppb for total AFs in corn intended for general animal consumption and two larger limits, 200 ppb and 300 ppb, for specific animals based on age and use [50]. The spatial analysis of mycotoxin contamination in storage scenarios is needed to reduce food waste. Previous research analyzing the spatial distribution of fumonisins in 3D in a stored grain pile showed that concentrations were greater at the base and edges of the pile [49]. The suggested reasons for this distribution were given as moisture settling near the base of the pile under the influence of gravity and favoring fungal development. The locations near the edge of the grain pile were under more aerobic conditions, allowing for greater fungal respiration. This is the reason for tracking CO_2_ and humidity levels in the current study, as CO_2_ and water are produced by respiration. Spatial differences in CO_2_ and moisture readings may indicate areas where more fungal respiration may be occurring. A real-time 12-sensor CO_2_ monitoring system developed for stored cereals showed increases in CO_2_ at a node where water was injected and at the nodes beneath it [51]. However, this work did not investigate general spatial patterns in mycotoxin build-up. Nevertheless, for a similar price to test one sample for aflatoxin, a single sensor could be re-used many times to detect grain spoilage.

This paper presents a proof-of-concept study investigating the spatial variation in the development of four mycotoxins in storage bins full of relatively clean and dirty corn, with respect to aflatoxin contamination, that were stored in different environmental conditions. As a preliminary investigation, this study aims to explore if, under a limited set of environmental conditions, toxin levels vary with depth in stored grain and between the center and edge of storage areas. Differences in toxin levels between three different temperature and RH combinations are investigated here for all corn samples and for the clean and dirty samples alone to determine which temperature and humidity levels should be studied in greater detail in the future. Aflatoxin is the main focus in this study due to the corn being clean or dirty with respect to its concentration. The importance of this preliminary spatial study relates to scenarios where testing could be prohibitively expensive such as in hot, humid developing countries where aflatoxin contamination is particularly rife. As a proof of concept, this research could demonstrate that consumption of certain areas of the grain piles or grain stored in silos should be avoided or could inform where to place one or a few sensors to detect the first signs of spoilage and how to use appropriate mitigation strategies. Determining risk zones within silos could also allow the grain to be divided into micro-batches for sale according to the location of the grain in the silo so that potentially less grain would be wasted.

The main research questions addressed in this study are as follows: (1) How do mycotoxin levels vary spatially within storage bins under different temperature and humidity conditions? (2) Are there significant differences in mycotoxin levels between clean and dirty corn samples, and between different temperature/humidity treatments? (3) Could the identification of high-risk zones within storage bins inform future targeted sampling strategies and mitigation efforts?

## 2. Results and Discussion

### 2.1. Environmental Conditions Within Bins

**Figure 1:**
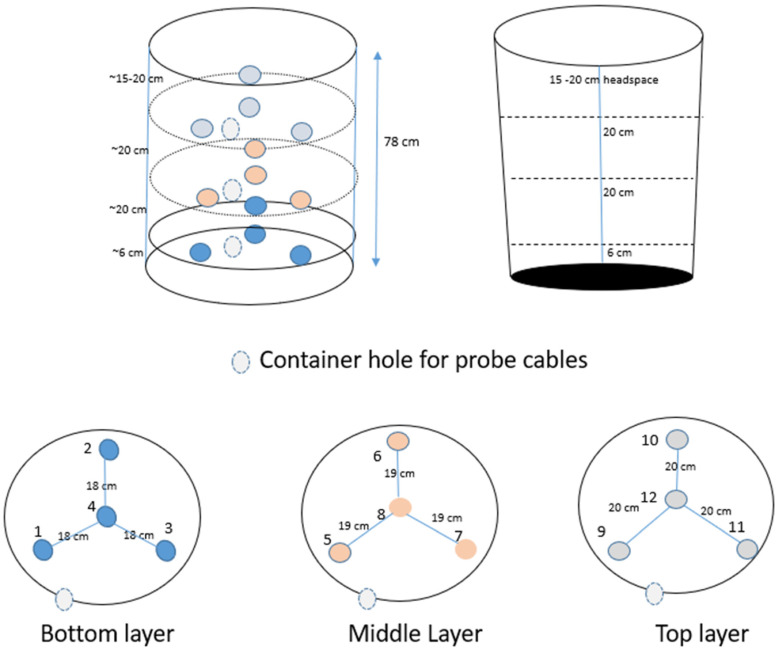
Diagram showing set-up of storage bins in environmental chamber.

Figure 1 shows the locations of CO_2_, temperature and humidity sensors within the corn bins. To determine if the sensors were giving sound readings, the sensors were left in empty buckets in the environmental cabinets for 48 h for each set of environmental conditions. For all environmental conditions, CO_2_ concentrations in the empty bins varied between 670 and 735 ppm. For the 21 °C, 30% RH treatment, temperatures were 1–2 °C higher than expected and the RHs were 6–7% higher than the settings in the environmental cabinet. For the 27 °C, 50% RH treatment, temperatures were 0.5–1.5 °C lower and the relative humidities were 3–4% higher than the environmental cabinet settings. For the 32 °C, 90% RH treatment, temperatures were 2–3 °C lower and the relative humidities were about 5% lower than the settings in the environmental cabinet. This suggests that particularly for the 32 °C, 90% humidity treatment, it was difficult for the high temperatures and humidities to equalize outside and inside the plastic bins.

**Figure 2:**
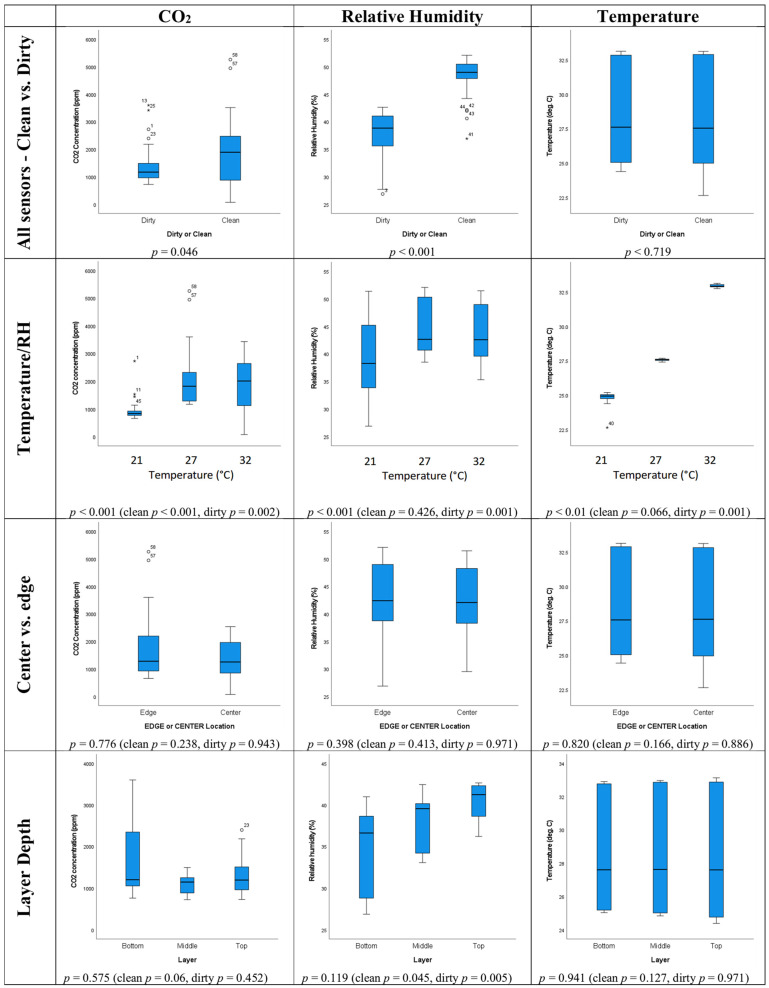
Environmental conditions in terms of average CO_2_ concentration, temperature and humidity within storage bins over study period. *p* values are for comparison tests using clean and dirty data together and *p* values in brackets are for clean and dirty data separately. Clean and dirty refer to very low or high concentrations of aflatoxin present in corn. * and hollow circles in the boxplots indicate values that are outliers (more than three standard deviations from the mean).

The top row of box plots in Figure 2 shows how the average environmental conditions for the study period varied between the clean and dirty corn bins based on all sensor locations for all three storage conditions. The CO_2_ levels and RHs were significantly (*p* < 0.05) higher in the clean rather than in the dirty corn. As respiration of fungi produces CO_2_ and water, one might expect the CO_2_ concentration and RH to be higher in the dirty corn. However, elevated CO_2_ levels might also be expected in the clean corn as mycotoxins other than aflatoxin are present, and the corn itself, which is more abundant in the bins than fungi, can respire. Also, corn at the bottom of the bin is under pressure from the weight of corn above, as well as at a higher humidity. This may be creating conditions at the bottom of the bin that could stimulate germination in the corn. The CO_2_ concentrations under all conditions when corn is present in the bin are higher (averaging 1000–2000 ppm) than the CO_2_ concentrations in empty bins (670–735 ppm), showing that the corn is respiring whether clean or dirty. Also, high CO_2_ levels in the dirty bin are likely to be localized around locations of contamination, so not all sensors will have the highest values.

The pattern of clean corn having higher CO_2_ levels may be different if the clean and dirty corn were compared separately for each temperature. The CO_2_ levels in the clean corn were low for the lower temperature and humidity treatment (21 °C, 30% RH), but very high in the clean corn for the 32 °C, 90% RH treatment. This is consistent with the findings that respiration rates in stored corn increased when stored at 75% RH as opposed to 65 or 70% RH [52]. In addition, it has been found that respiration of corn depends on both moisture content and temperature [53]. As high aflatoxin concentrations of the dirty corn have been associated with drought conditions in the field [27], the moisture content of the clean corn is likely to be larger, favoring greater respiration and therefore higher RH and CO_2_ levels. As a large volume of corn will be respiring all the time, a temporal analysis of spikes in CO_2_ levels or sudden increases for individual sensors rather than looking at general CO_2_ levels may be more helpful in identifying CO_2_ from fungal respiration.

The last box plot on the top row of Figure 2 shows that for the range of temperatures in the clean and dirty corn, the temperatures measured by the sensors were quite consistent and there was not a significant difference in the temperatures between clean and dirty corn. This is expected given that the bins of clean and dirty corn were stored in the same environmental chamber and no appreciable heat production is expected from fungal respiration.

The second row of box plots in Figure 2 shows how the environmental conditions varied between the different temperature/RH treatments for clean and dirty corn when considered together. Box plots plotted separately for the clean and dirty corn showed very similar patterns to the clean and dirty corn considered together, so they are not shown, but *p* values for the associated comparison tests performed are shown. The CO_2_ levels and RHs were significantly (*p* < 0.05) lower for the 21 °C, 30% RH treatment than for the other two temperature/RH treatments. This is expected as the dirty corn was known to be contaminated with aflatoxin, which is produced by *Aspergillus* fungi whose optimal conditions for growth and aflatoxin production are between 30 °C and 37 °C (Table 1). This pattern is also expected for the clean corn due to the difference in RH between temperature treatments. However, the difference in RH between the temperature treatments is less pronounced for the clean corn (*p* = 0.426). The similarity in the RH readings for the 27 °C, 50% RH and 32 °C, 90% RH environments is surprising but consistent with the RH values for the empty bins which were higher than expected for the former and lower than expected for the latter. This meant that conditions within the corn for these two treatments ended up being very similar. It is likely difficult for the extremely humid air to diffuse fully into the stored corn in a plastic bin within the environmental cabinet.

The last box plot on the second row of Figure 2 shows that the temperature conditions within both bins were consistent and narrow (*p* < 0.01), yet the temperatures detected at the sensor nodes were slightly higher than expected in each case. For example, the corn stored at 21 °C should have measured 21 °C at the sensor, but Figure 2 shows that the temperature was around 25 °C. For the 27 °C treatment, the temperature within the grain bins should have been 27 °C but it was around 27.5 °C, and for the 32 °C treatment, it should have been 32 °C, but was about 33 °C. This is likely a result of the solid corn heating up more than the air. Also, the temperature sensors are accurate to ±1 °C and were placed in the corn on wire mesh trays. As the wire trays are good conductors, they could heat up to slightly higher temperatures than the corn, but this effect is likely to be marginal. Finally, the temperature results are largely consistent with the readings from running the sensors in empty bins. This showed that the temperatures for the 21 °C and 27 °C empty bins were 1–3 °C higher than the environmental cabinet settings, but the sensors suggested that the temperature in the 32 °C empty bin was 2–3 °C cooler than expected.

The third row of box plots in Figure 2 shows how the how the environmental conditions varied between the central and edge locations within the bins. There were no significant differences in CO_2_, humidity or temperature observed between central and edge locations. The distributions of humidity and temperature measurements between central and edge locations were very similar. There was a slightly higher mean and range of CO_2_ values in the edge locations, but this was not significant at *p* = 0.05. Nevertheless, the smaller *p*-value for the clean corn shows that this effect was more evident in the clean corn. Following on from previous work relating to a grain pile, higher CO_2_ values in edge locations are expected if there is more aeration at the edges of the bin, allowing for more respiration in general in edge locations [49]. However, this effect was slight in the current experiment as these bins had solid plastic walls, apart from one hole per layer to allow the wires from the sensors to be connected to a computer.

The final row of box plots in Figure 2 shows the differences in environmental conditions between layers in the grain bins. Although the median values of CO_2_ are similar between layers (*p* = 0.575), the range of values is greatest for the bottom layer and next greatest for the top layer. This is likely due to more respiration taking place with any liquid moisture present in the bins falling under the influence of gravity to the bottom layer and more respiration in the top layer due to greater aeration within the headspace at the top of the bin (Figure 1). The *p*-values for RH were smaller (*p* = 0.119, clean and dirty; *p* = 0.045 clean) and highly significant (*p* = 0.005) for dirty corn alone. The box plot shows observable differences in the median and range of humidity for each layer in the bins, with the bottom layer having the lowest and the top layer having the highest RH. This is expected as hot air rises and hot air can hold more moisture in the form of vapor than cold air can. This also might mean that where cold air is sinking, water vapor may be changing state into liquid water and favoring respiration in the bottom layers, as suggested previously [49].

### 2.2. Aflatoxin

#### 2.2.1. Summary Statistics and Spatial Distribution

Table 2 shows the initial average concentrations for two separate thoroughly mixed bins of corn with the averages being based on the concentrations of 12 randomly selected samples from each bin that were mixed together. Aflatoxin in the clean and dirty corn was 0 and 300 ppb, respectively. This shows that initial aflatoxin concentrations were markedly different in the clean and dirty corn. Also, the average concentration of the dirty corn was at the highest FDA limit for aflatoxin and the whole batch would be discarded as not even being fit for use by old cattle and swine [50] or could be sold at a far lower price to the biofuels industry to be fermented into ethanol.

Table 3 shows the summary statistics for aflatoxin in each dataset following storage for two months at the given temperature and humidity settings mentioned in the Section 4. By the end of the storage period, the aflatoxin levels in the clean and dirty corn had increased but those for the clean corn were still low (<4.5 ppb). In contrast, the aflatoxin levels for the dirty corn had approximately doubled at all temperatures, and the maximum values represented about 10 times the FDA 300 ppb limit (>3000 ppb). When separated by temperature/RH treatment, it is clear that storage for 2 months at 21–32 °C and differing humidities increases the mean values of aflatoxin, with the largest mean (697 ppb) being for the 27 °C, 50% RH treatment and the least for the 32 °C, 90% RH treatment (547 ppb) (Table 3). The maximum values following storage are highest for the corn stored at 32 °C and 90% RH (3200 ppb). The similarity between the 27 °C, 50% RH and 32 °C, 90% RH treatments in terms of aflatoxin concentrations may be expected given that the sensors suggest that conditions inside the 32 °C bin of corn were cooler and less humid than in the rest of the environmental chamber and the conditions in the 27 °C bin were warmer and more humid than in the rest of the environmental chamber.

The mean and maximum aflatoxin values for the dirty corn were far higher than the 20 ppb FDA limit for general consumption food and the 300 ppb threshold allowable for finishing cattle [50]. The 32 °C, 90% RH data had the largest coefficient of skewness, showing that the very highest values were rarer than in the other datasets. The aflatoxin values for the clean corn stored in all conditions are very low, with a maximum of 4.5 ppb and a mean of 0.38 ppb. The skew of 2.76 is due to most of the aflatoxin values for the clean corn being zero. In contrast, the dirty corn stored in all conditions contains values of up to 3200 ppb and a mean of 1195 ppb, with a skew value of 0.69, showing a relatively normal distribution. This would mean that all the dirty corn would need to be disposed of or be sold as biofuel and should not be sold by the farmer for human or animal consumption as the average aflatoxin concentration exceeds all FDA thresholds [50].

**Figure 3:**
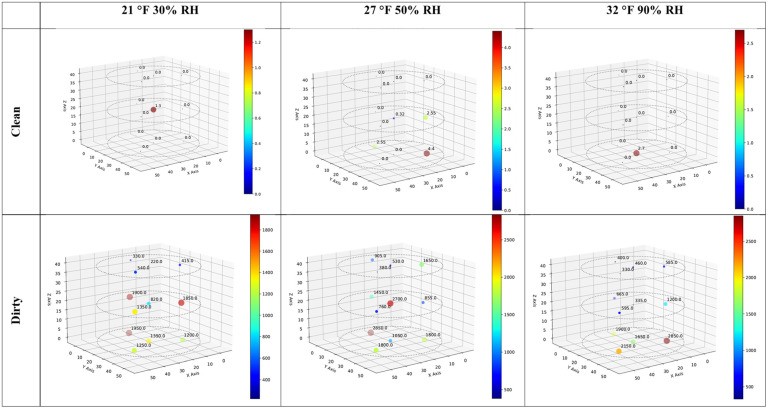
Three-dimensional plots showing the spatial distribution of aflatoxin (ppb) build-up in clean and dirty corn stored under three temperature/RH combinations (21 °C, 30% RH; 27 °C, 50% RH; and 32 °C, 90% RH). *Clean and dirty refer to very low or high concentrations of aflatoxin present in the corn. Each colored dot shows the average toxin concentration of two samples*.

Figure 3 shows the spatial distribution of average aflatoxin levels (the average of two values) at sensor locations for clean and dirty corn after being stored at 21 °C, 30% RH; 27 °C, 50% RH; and 32 °C, 90% RH for two months. For all the clean corn, most values are zero, apart from some low values in the middle and bottom layers of the bin. For the dirty corn, values in the lower two layers tend to be >1000 ppb, apart from a few values for the middle layer in the 32 °C, 90% RH bin that are <1000 ppb. The values for the top layers of each bin are generally <600 ppb, apart from two values >900 ppb in the top layer of the 27 °C, 50% RH bin. Figure 3 shows two elevated values in central locations for the clean corn, one in the 21 °C, 30% RH bin’s middle layer and one in the 32 °C, 90% RH bin’s bottom layer. However, for the 27 °C, 50% RH sample, both of the non-zero values are at edge locations. For the dirty corn, there is a slight tendency toward lower values in the central locations for the 21 °C, 30% RH and 32 °C, 90% RH conditions, which agrees with previous findings [49] and the hypothesis that there is less mycotoxin build-up in central locations than edge locations because edge locations are more aerobic. However, the same pattern was not found for the 27 °C, 50% RH conditions.

#### 2.2.2. Layer Depth

**Figure 4:**
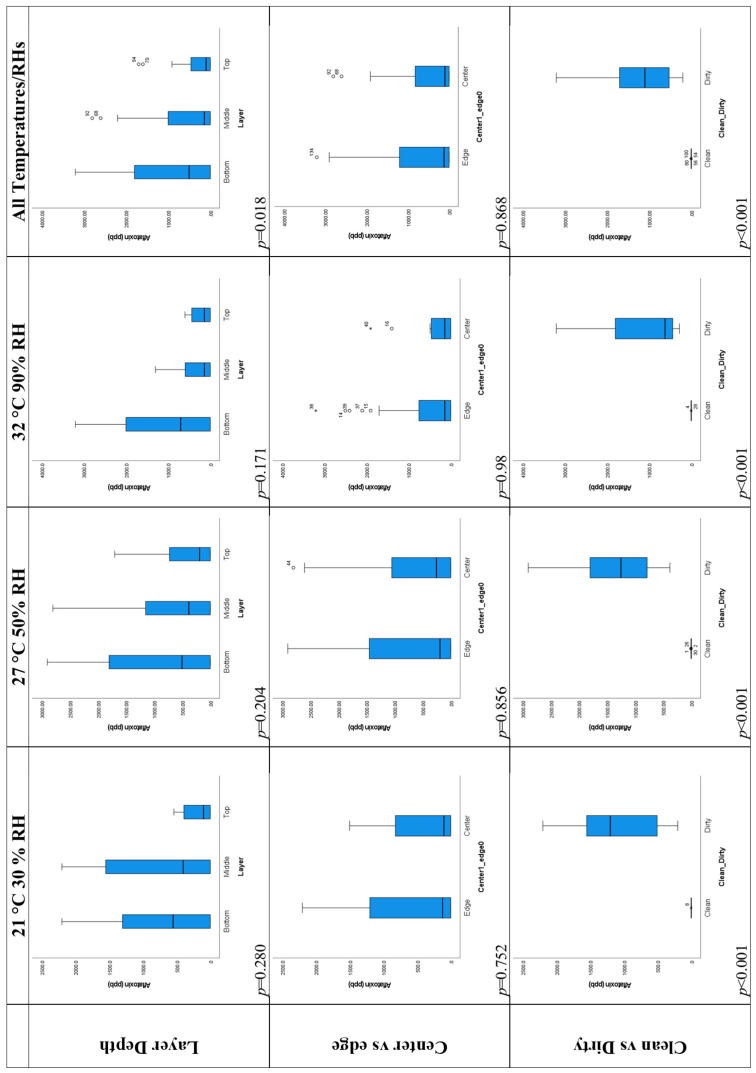
Box plots showing the comparison of aflatoxin levels (ppb) between different layers, center and edge locations, and clean and dirty corn stored under different temperature/RH combinations (21 °C, 30% RH; 27 °C, 50% RH; and 32 °C, 90% RH). Clean and dirty refer to very low or high concentrations of aflatoxin present in the corn. * and hollow circles in the boxplots indicate values that are outliers (more than three standard deviations from the mean).

The top row of box plots in Figure 4 shows differences in the distribution of aflatoxin levels in the layers for each temperature/RH treatment and for all treatments considered together. For each treatment, the aflatoxin levels in the top layer are lowest. For the 27 °C, 50% RH and 32 °C, 90% RH treatments, the bottom layer has the highest aflatoxin levels. The box plots show the expected general trends given previous findings [49] that mycotoxin levels tend to be greatest near the base of a grain pile, probably due to increased liquid moisture collecting there under the influence of gravity, and this is a significant difference (*p* = 0.018) when the data for all temperature/RH treatments are considered together.

**Figure 5:**
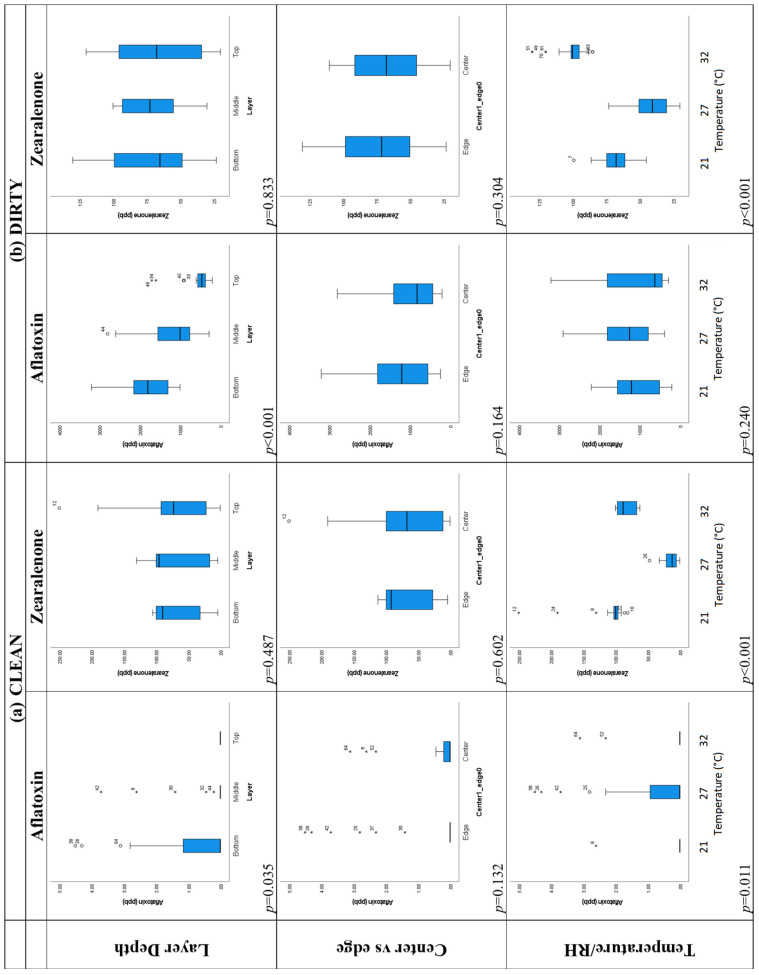
Box plots showing aflatoxin and zearalenone levels between different layers, center and edge locations, and different temperature/RH combinations (21 °C, 30% RH; 27 °C, 50% RH; and 32 °C, 90% RH) for (**a**) only clean and (**b**) only dirty stored corn. Clean and dirty refer to very low or high concentrations of aflatoxin present in the corn. * and hollow circles in the boxplots indicate values that are outliers (more than three standard deviations from the mean).

However, when the different temperature/RH treatments are considered separately, the box plots show the expected trends, but the differences in aflatoxin levels between the layers are not significant at *p* = 0.05 (Figure 4 and Table S1). The difference in the layers is significant (*p* = 0.035 and *p* < 0.001, respectively) when the clean and dirty corn are considered separately (Figure 5 and Table S1). The layers also show the expected order of aflatoxin concentrations given the hypothesis of higher concentrations occurring in more moist locations near the base of the bin where moisture collects under the influence of gravity. Table 4 shows that the mean aflatoxin concentration in the top layer for the 21 °C, 30% RH treatment was 188 ppb. This is below the FDA 200 and 300 ppb limits, but for the other layers, the average aflatoxin concentrations were 740 and 719 ppb, which are well above the FDA limits [50]. In this situation, it would be possible for the farmer to gain more income for the top layer of stored grain rather than dispose of/sell all layers of grain for a far lower price.

#### 2.2.3. Central and Edge Locations

The second row of box plots in Figure 4 shows that there are higher levels of aflatoxin in edge locations compared to central locations for each temperature regime and for all temperatures considered together. This is the pattern that would be expected given the hypothesis that more aflatoxin builds up in more aerobic edge locations than in central locations. However, all *p*-values are far from significant at *p* = 0.05 (Figure 4 and Table S1). The *p*-values for differences in aflatoxin between central and edge locations are much smaller when the clean and dirty corn are considered separately (see Figure 5, *p* = 0.132 and *p* = 0.164, respectively). The large *p*-values when clean and dirty corn are considered together are probably the result of almost opposite patterns being shown in the clean and dirty corn, with central locations having higher aflatoxin levels in the clean corn and lower aflatoxin levels in the dirty corn (see Table 4). The levels of aflatoxin are so low in the clean corn that they can hardly be considered to have markedly higher levels in central locations. All levels are well below FDA thresholds for the clean corn (see Figure 5—left column of box plots). While there were marked differences in aflatoxin concentration between central and edge locations in the grain which were sometimes significant, the differences were not marked enough to have areas of the grain with some concentrations below and others above FDA thresholds. All concentrations were above the 300 ppb FDA threshold (Table 4). However, for grain piles, there is a far greater difference in the amount of aeration between central and edge locations. This effect needs to be tested in future for grain piles.

#### 2.2.4. Clean and Dirty Corn

The clean and dirty corn was selected based on there being appreciable aflatoxin levels in the dirty corn and low-to-no aflatoxin in the clean corn. Therefore, highly significant differences (*p* < 0.001) in the aflatoxin levels between the clean and the dirty corn after each temperature/RH treatment were expected (see Figure 4 and Table S1). In each case, the aflatoxin levels were significantly higher in the dirty corn, as can be seen from the minimum, maximum, and mean values reported in Table 3.

#### 2.2.5. Temperature/Relative Humidity

The final row of box plots in Figure 5 shows the effect of temperature/RH treatment when clean and dirty corn were considered separately. For the clean corn, the 21 °C, 30% and 32 °C, 90% RH treatments had median aflatoxin concentrations of 0 ppb. The 27 °C, 50% RH treatment had a larger interquartile range and several large outliers. For the dirty corn, the 32 °C, 90% RH had the largest range of values but lowest median whereas the 21 °C 30% RH treatment had the smallest range and interquartile range The reasons for this result are likely related to the optimum temperatures for fungal growth and aflatoxin production (Table 1). Fungal growth may be more likely at 27 °C than at 32 °C, but aflatoxin production is greatest at the highest temperatures (>37 °C, Table 1, [19]). The differences in RH level between the different temperature/RH treatments are also important in determining fungal growth and aflatoxin production. Given the difficulty in relating the RH environmental levels to ERH, it can only be surmised that the water content of the grain was relatively low compared to the RH of the air in the stored corn and that at 21 °C and 30% RH and 27 °C and 50% RH, some moisture may have left the corn and aided fungal growth, allowing for the high aflatoxin levels observed in these lower temperature treatments. Another potential reason for the lack of difference between the 32 °C, 90% RH and 27 °C, 50% RH treatments is that the sensors suggested that the highest temperatures and humidities in the environmental chamber were not penetrating the bucket and diffusing fully through the corn; so, the environmental conditions within the corn for these two treatments ended up being quite similar.

Table S4 shows that when clean and dirty corn are considered together, there is not a significant difference in the aflatoxin levels for different temperature/RH treatments (*p* = 0.429) and that the temperatures do not show the expected order for aflatoxin levels, with the lowest temperature/RH having the lowest levels and the highest temperature having the highest aflatoxin levels. When the clean corn is considered alone, there is a significant difference (*p* = 0.011) in aflatoxin levels between temperature treatments, but the levels are highest in the 27 °C treatment. When the dirty corn is considered alone, the difference between temperatures is not significant (*p* = 0.240). This is because the 32 °C, 90% RH treatment had the lowest mean aflatoxin concentration while having the largest range of aflatoxin values (Table 3). Based on temperature alone, aflatoxin levels might be expected to be highest for the 32 °C treatment as it is closest to 30 °C and 37 °C, the optimal temperatures for *Aspergillus* fungal growth and aflatoxin production [19]. However, as the RH levels also varied with temperature in this experiment and ERH is unknown throughout the corn, the effects of the humidity and temperature are being confounded. This may explain the lack of significant difference in aflatoxin levels between temperature/RH treatments for the dirty corn. This confirms the future necessity of investigating a range of RH settings for each temperature and replicating each temperature/RH combination.

### 2.3. Deoxynivalenol (DON)

Table 2 shows that the average deoxynivalenol levels from 12 randomly selected samples in the thoroughly mixed clean and dirty corn prior to storage at different temperatures and humidities were very similar (28 and 29 ppb, respectively). The graphs and tables associated with the spatial distribution of deoxynivalenol in the storage bins under different temperature/RH conditions are shown in the Supplementary Materials, Figures S1 and S2 and Tables S3 and S4. The results for this toxin are reported in the Supplementary Materials because most values were zero for the 21 °C, 30% RH and 32 °C, 90% RH treatments and the maximum values across all treatments were only 0.4 ppb. This shows that each set of storage conditions leads to a reduction in deoxynivalenol concentration over time. Given the low values of deoxynivalenol for each treatment and their limited variability, no significant differences between layers or central and edge locations were observed (Figure S2 and Table S4). The only criterion which showed significant differences (*p* < 0.001) in concentrations was temperature/RH treatment, with the 21 °C, 30% RH treatment always having the lowest concentrations and the 27 °C, 50% RH treatment having the highest (Table S4). There was also a significant (*p* < 0.001) difference between the clean and dirty corn for the 27 °C, 50% RH treatment. This is likely because the optimum temperatures for *Fusarium graminearum* mold growth and deoxynivalenol production are 25 °C and 20–30 °C, respectively, both of which are close to, or include, 27 °C (Table 1 [16]).

### 2.4. Fumonisin

#### Summary Statistics and Spatial Distribution

Table 2 shows that the initial average fumonisin concentrations from 12 randomly selected samples from thoroughly mixed clean and dirty corn were similar, being 3.46 and 5.21 ppm, respectively. However, following storage at 21–32 °C and 30–90% RH, the mean concentrations were slightly lower, being 2.87, 3.08 and 1.14 ppm for 21 °C, 30% RH; 27 °C 50% RH; and 32 °C, 90% RH, respectively (see Supplementary Materials, Table S6). Due to the low initial levels of fumonisin in the clean and dirty corn and due to the drop in mean concentrations following storage, fumonisin is not examined in detail here, but graphs and tables of the results can be found in the Supplementary Materials, Figures S3–S5 and Tables S5 and S6.

The figures and tables in the Supplementary Materials show significant differences in the concentrations between layers when all temperature/RH conditions are considered together (*p* = 0.038) and for the 21 °C, 30% RH treatment (*p* = 0.003), with the bottom layer having the highest concentrations in both cases. Also, when the clean corn was considered alone, there were significantly higher concentrations in the bottom layer (*p* = 0.003). The layers showed the expected order of concentrations based on previous work [49], with the bottom layer having the highest concentrations and the top layer having the lowest concentrations. For some treatments, it was evident that the middle layer had the lowest fumonisin concentrations (Table S5). This pattern may be a result of greater aeration near the top layer due to the headspace (Figure 1), as previous work hypothesized that mycotoxin concentrations would be higher in more aerated locations as fungi need oxygen to respire [49].

When the mean fumonisin concentrations were compared between layers for each temperature/RH treatment, Table S5 shows that the mean concentrations in the top and middle layers for the 21 °C, 30% RH treatment were 1.577 ppm and 2.267 ppm, respectively, which are both below the FDA 4000 ppb (4 ppm) limit. Meanwhile, for the bottom layer, the average fumonisin concentration was 4.763 ppm, which is above the FDA limit (Table 1 [54]). This means that in this situation, it would be possible for the farmer to gain more income for the top two layers of stored grain rather than dispose of or sell all layers of grain for a far lower price.

There were generally no significant differences in fumonisin concentration between central and edge locations, but the fumonisin concentration was significantly higher (*p* = 0.006) at the edge for the 27 °C, 50% RH treatment. Table S5 shows that concentrations in center and edge locations were below the 4000 ppb FDA threshold (Table S5).

For the dirty corn, there was a significant difference between temperature treatments (*p* < 0.001). The lowest concentrations for clean and dirty corn were for the 32 °C, 90% RH treatment, but the highest concentrations were found for the 21 °C, 30% RH-treated clean corn. This is because the optimum temperature for fumonisin build-up is 22 °C (Table 1, [18]).

### 2.5. Zearalenone

#### 2.5.1. Summary Statistics and Spatial Distribution

**Figure 6:**
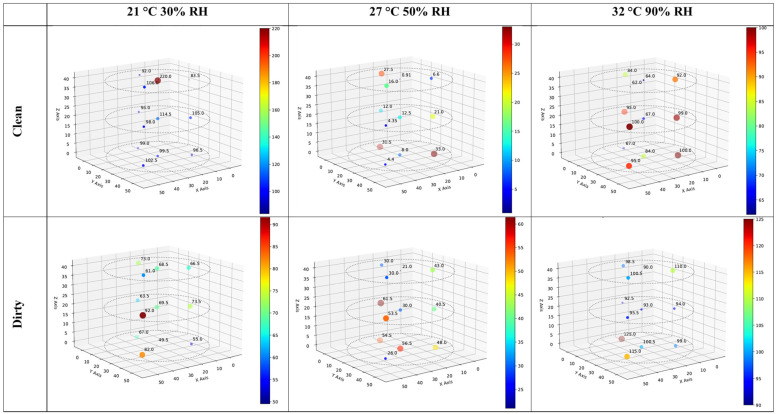
Three-dimensional plots showing the spatial distribution of zearalenone (ppb) build-up in clean and dirty corn stored under three temperature/RH combinations (21 °C, 30% RH; 27 °C, 50% RH; and 32 °C, 90% RH). Clean and dirty refer to very low or high concentrations of aflatoxin present in the corn. Each colored dot shows the average toxin concentration of two samples.

Table 2 shows that the initial average concentrations of zearalenone from 12 randomly selected samples in thoroughly mixed clean and dirty corn were markedly different, being 22 and 115 ppb, respectively. Table 5 shows that the maximum concentrations following different temperature/RH treatments are far higher for clean and dirty corn (250 ppb and 130 ppb, respectively). Also, the highest mean and maximum values were found for the 21 °C, 30% RH and 32 °C, 90% RH treatments, with the concentrations for the 27 °C, 50% RH treatment being markedly lower. Figure 6 shows the spatial distribution of zearalenone values in the bins following different temperature/RH treatments. The values are lowest in general for the 27 °C, 50% RH treatment and higher for the 21 °C, 30% RH and 32 °C, 90% RH treatments, but there is not a great difference between the clean and dirty corn. Also, the only treatments that seem to show larger concentrations in the lower layers are the 27 °C, 50% RH and 32 °C, 90% RH treatments for dirty corn.

#### 2.5.2. Layer Depth

**Figure 7:**
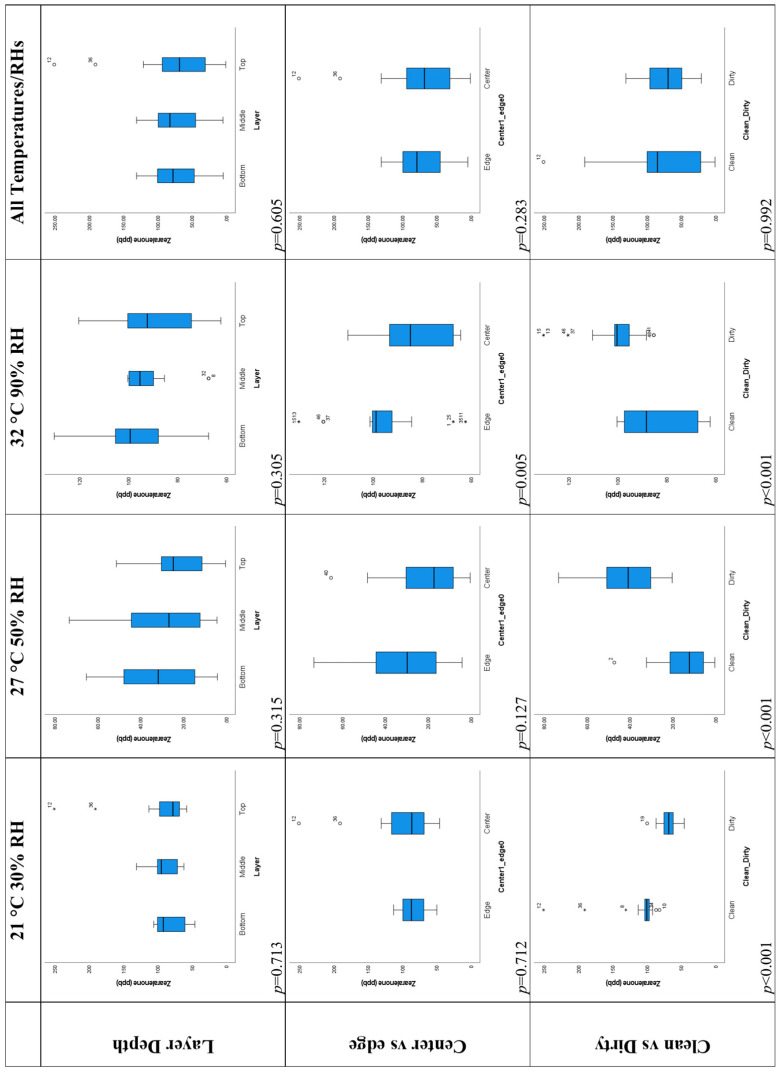
Box plots showing the comparison of zearalenone levels (ppb) between different layers, center and edge locations, and clean and dirty corn stored under different temperature/RH combinations (21 °C, 30% RH; 27 °C, 50% RH; and 32 °C 90% RH). Clean and dirty refer to very low or high concentrations of aflatoxin present in the corn. * and hollow circles in the boxplots indicate values that are outliers (more than three standard deviations from the mean).

The top row of box plots in Figure 7 shows how zearalenone concentration varies with layer depth. For all temperature/RH treatments, there is no significant difference between the layers (*p* = 0.305–0.713). However, for the dirty corn and some temperature/RH treatments, the order of concentrations between layers shows the expected pattern suggested by previous research [49], with the bottom layer having the highest concentrations and the top layer having the lowest concentrations for the 27 °C, 50% RH treatment, the 32 °C, 90% RH treatment and all temperature/RH treatment data (Table 4). However, differences between layers were not significant (*p* = 0.487 and *p* = 0.833 for clean and dirty corn, respectively). As there is currently no FDA limit for zearalenone, it is not possible to determine based on the mean concentrations by layer (Table 4) if some grain waste could be avoided by treating the grain stored in different layers as different micro-batches. However, this is unlikely given that the differences between layers were not significant.

#### 2.5.3. Central and Edge Locations

The middle row of box plots in Figure 7 shows the difference in zearalenone concentration between central and edge locations. The 27 °C, 50% RH and 32 °C, 90% RH treatment and all temperature/RH treatment data show higher levels of zearalenone in edge locations compared to central locations, as expected given previous work [49], but the difference was most marked and only significant for the 32 °C, 90% RH data (*p* = 0.005, Table 5). This may be due to the RH and temperature within the 32 °C, 90% RH environmental chamber not diffusing well into the bin and through the corn. The same pattern was shown with lower values in central locations and higher concentrations at the edges when the clean and dirty corn were considered separately, but the differences were not significant (*p* = 0.602 and *p* = 0.712).

One limitation of this study is that we did not directly measure the equilibrium relative humidity (ERH) of the corn, which is a key factor in determining the growth and mycotoxin production of fungi in stored grain. ERH is the humidity at which the corn kernels neither gain nor lose moisture, and it can differ from the RH of the air surrounding the grain. The relationship between air RH and grain ERH is complex and depends on factors such as temperature, moisture content and the presence of other substances in the grain. By relying on the RH measurements of the air within the bins, we may not have fully captured the moisture conditions experienced by the corn at a kernel level. Future studies could benefit from incorporating methods to directly measure or estimate the ERH of the corn, such as using humidity probes inserted into the grain or calculating ERH based on grain moisture content and temperature data.

As there is currently no FDA limit for zearalenone, it is not possible to determine based on the mean concentrations between central and edge locations (Table 4) if some grain waste could be avoided by treating the grain stored in central and edge locations as different micro-batches. However, as concentrations were significantly different for the 32 °C, 90% RH data only between central and edge locations, it seems that the degree of aeration is a more important factor in zearalenone build-up than for aflatoxin and fumonisin, and treating edge and central locations as separate micro-batches might be a successful strategy for reducing wasted grain. This needs further testing.

#### 2.5.4. Clean and Dirty Corn

There were significant differences (*p* < 0.001) between the clean and dirty corn for each temperature/RH treatment. However, when all temperature/RH treatments were considered together, the difference between the clean and dirty corn was not significant (*p* = 0.992). This shows that the pattern for the 21 °C, 30% RH data was the opposite of that for the 27 °C, 50% RH and 32 °C, 90% RH data. For the latter two temperature/RH combinations, the dirty corn had higher zearalenone concentrations. This is likely because the optimum temperature for *Fusarium graminearum* and *Fusarium culmorum* fungal growth is close to 27 °C and the temperature for optimal zearalenone build-up is higher than 27 °C (Table 1, [17]). The higher zearalenone concentrations for the 27 °C, 50% RH and 32 °C, 90% RH treatments are also due to the environmental conditions within the corn for these two treatments being quite similar.

#### 2.5.5. Temperature/Relative Humidity

When a comparison was made between different temperature/RH treatments for all corn and for clean and dirty corn separately, the differences between temperature/RH treatments was highly significant (*p* < 0.001) for zearalenone. In each case, the temperature/RH treatment with the lowest concentrations was 27 °C, 50% RH. For all data and the dirty corn, the 32 °C, 90% RH treatment showed the highest zearalenone concentrations, but for the clean corn, the 21 °C, 30% RH treatment had the highest concentrations. The 32 °C, 90% RH treatment having the highest concentrations likely relates to the optimum temperature for zearalenone production being >27 °C, but the medium concentrations for the 21 °C data suggest that the low RH of air in the corn at this temperature may have caused some moisture to come from the corn and contribute to fungal growth. More levels of humidity need to be investigated in the future and replicated.

### 2.6. Summary

#### 2.6.1. Layer Depth

For aflatoxin, there were significant differences (*p* = 0.05) between the layers when all temperatures were considered together and when clean and dirty corn were considered separately, with the top layer having the lowest and the bottom layer having the highest concentrations. Zearalenone showed the same patterns with depth, but the differences between layers were not significant at *p* = 0.05. Fumonisin showed some significant differences in concentrations between layers, but the pattern was slightly different, with the middle layer showing the lowest concentrations, suggesting that this toxin may respond more to aeration as the top layer had some headspace. Deoxynivalenol showed no effect of layer depth as the concentrations for all temperatures were so low.

#### 2.6.2. Central and Edge Locations

Aflatoxin levels showed the expected higher concentrations in edge locations, but the differences were not significant at *p* = 0.05. For fumonisin and zearalenone, the 27 °C, 50% RH and 32 °C, 90% RH treatments showed higher concentrations in edge locations, butthe difference was only significant at *p* = 0.05 for the 27 °C, 50% RH treatment for fumonisin and the 32 °C, 90% RH treatment for zearalenone.

#### 2.6.3. Clean and Dirty Corn

As might be expected given that the clean and dirty corn was originally selected based on known aflatoxin contamination or lack thereof, the aflatoxin concentrations after the different temperature treatments were all significantly higher (*p* = 0.05) in the dirty corn. For zearalenone, there were differences that were temperature/RH-dependent, with some temperature/RH combinations showing higher concentrations in the clean corn.

#### 2.6.4. Temperature/Relative Humidity

For aflatoxin, there were no significant differences between the temperature/RH treatments at *p* = 0.05; however, the 27 °C, 50% RH treatment had the highest mean concentrations and the 32 °C, 90% RH treatment had the highest maximum concentrations. Fumonisin and zearalenone showed significant differences (*p* = 0.05) in concentration between temperature treatments. For fumonisin, the 32 °C, 90% RH treatment had the lowest concentrations, whereas for zearalenone, the 32 °C, 90% RH treatment had the highest concentrations. For deoxynivalenol, there were significant differences (*p* = 0.05) based on temperature/RH combinations, with the 27 °C, 50% RH treatment showing the highest concentrations.

## 3. Conclusions

This study has shown that there is a tendency for several mycotoxins to show patterns of contamination associated with storage depth. This agrees with the theory that fungi develop in moist locations and that liquid water falls under the influence of gravity to lower layers in storage settings. This study also showed higher levels of toxin build-up in edge locations compared to central locations, but the differences between edge and central locations tended not to be significant. The differences between layers and central and edge locations were also affected by whether the corn was clean or dirty and the temperature/RH regime it had been stored under. When the clean corn was investigated at a temperature that did not tend to increase the concentration of a given toxin, the concentrations were generally too low to detect significant differences between the layers or between central and edge locations.

The findings of this study agree with previous work [49] and suggest that it might be possible to divide stored grain into high- and low-risk zones for given mycotoxins based on layer depth, likely moisture accumulation and degree of aeration. Once low- and high-risk zones have been defined, if the average concentration of a mycotoxin is over legislative limits, concentrations for the high- and low-risk zones could be measured separately and perhaps only the grain from the high-risk zones would need to be discarded or sold to the biofuel industry. This would result in less grain waste. Less grain waste is needed if the increasing global population is to be safely and properly fed in a warmer future. Should further spatial studies show similar results, it may be worth altering the standard procedures for sampling grain to take into account likely patterns in spatial variation of contamination levels rather than ignore this concept and assume all variation is random.

The findings of this study could also help those in less developed countries where little testing is performed to determine which grain is most and least safe to eat. This initial study suggests that it is worth investigating more temperature and humidity combinations and replicating conditions to test the consistency of the results. Examination of the different levels of humidity will be crucial as it is difficult to know the ERH in storage settings, and the sensors showed that the highest temperature and RH conditions may not penetrate fully into densely packed corn in a bin or silo. Most previous work on the conditions under which various fungi thrive and produce mycotoxins has been completed in laboratory settings where ERH is known.

This initial study, which examined the spatial distribution of mycotoxins under a range of temperature and RH conditions, provides compelling evidence that spatial analysis of mycotoxin build-up in storage settings is a promising avenue for further research. The finding that the build-up of toxins is not random but shows spatial patterns has key implications for how foodstuffs are sampled to determine whether regulatory threshold concentrations are exceeded. The findings can guide future research by identifying the most relevant temperature and RH levels for each mycotoxin, ultimately leading to improved sampling strategies and targeted mitigation efforts to reduce food waste and enhance food safety.

## 4. Materials and Methods

### 4.1. Experimental Set-Up

One reason spatial mycotoxin studies are so rare is due to the number of samples that need to be analyzed at significant expense and due to the difficulty in fully controlling and replicating the results. Due to the limited volume capacity and availability of environmental cabinets, only two bins of corn could be stored in one environmental cabinet at a time. An EGC C6 Environmental Control System managed with the ECoSys Software ECoSys Central Control and Monitoring System (CCMS) (Standard Edition R2) (Environmental Growth Chambers, Chagrin Falls, OH, USA) was used to carry out the storage studies. The chamber was programmable from 0 °C to 50 °C with a ±0.5 °C uniformity and adjustable from 2 to 95% RH, subject to dew point restrictions.

Two 32-gallon plastic storage bins were set up as shown in Figure 1. Each bin contained corn that was known to be relatively clean or dirty with respect to aflatoxin contamination. The commercially available corn hybrid Dekalb VT2PRO (DKC 65–93) was planted in 2021 to produce the corn samples used in this study. Clean corn (or corn with low levels of aflatoxin contamination) was planted and harvested in a well-managed Field 1C at the Delta experimental station in Stoneville, MS, while the dirty (high levels of aflatoxin contamination) corn was planted and harvested in a field known to have a high degree of aflatoxin contamination (Field #3) at the Delta experimental Station, MS. Additional corn produced in Tifton, GA, was mixed (1:1 ratio) with the high-aflatoxin-contaminated VT2PRO samples. The corn from Tifton, GA, had received an artificial inoculation with spores of the toxicogenic isolate NRRL 3357. 

The bin with clean corn was considered the control to compare mycotoxin and environmental conditions for the dirty corn with. Nevertheless, the initial concentrations of three mycotoxins other than aflatoxin in the bin of clean corn were >0 ppb (Table 2). Due to the initial concentrations of deoxynivalenol and fumonisin being similar in the clean and dirty corn, detailed graphs and statistical analysis for these toxins are provided in the Supplementary Materials. The main focus here is on aflatoxin and zearalenone due to the marked differences in the initial concentration of these toxins in the clean and dirty corn.

Each storage bin contained 12 wired sensors that tracked temperature (0–55 °C ± 1 °C accuracy), humidity (0–95% ± 5% accuracy) and CO_2_ levels from 0 to 10,000 ppm with an accuracy ± 50 ppm or 3% of the reading (Cozir, CM-0199, CO_2_ METER, Ormond Beach, FL, USA). The sensors were arranged in three layers within the bin (top, middle and bottom) on wire mesh trays with 20 cm of space between layers, as shown in Figure 1. Each layer of sensors contained four sensors which were configured and spaced as shown in Figure 1, with one sensor in the center and three around the edges of the layer. Figure 1 also shows the location of the holes in the bin wall that were necessary to attach the cables from the sensors to a laptop computer for tracking environmental conditions within the corn in each bin.

It is important to note that the sensors measured the RH of the air within the storage bins, which is different from the equilibrium relative humidity (ERH) of the corn itself. ERH is the humidity at which the corn kernels neither gain nor lose moisture, and it is a crucial factor in determining the growth and mycotoxin production of fungi in stored grain. The ERH of corn is influenced by factors such as moisture content, temperature and the presence of other substances in the grain. In this study, we did not directly measure the ERH of the corn due to technical limitations, and instead relied on the RH measurements of the air within the bins as a proxy for the moisture conditions experienced by the corn.

Both bins, one with clean corn and the other with dirty corn, were stored in one environmental chamber per constant temperature and RH combination for a period of two months (60 days). The sensors measured and recorded temperature, humidity and CO_2_ concentration every 30 min over the two-month period. The values of CO_2_ from the sensors that were zero or less than the limit of detection were excluded from the analysis as the current CO_2_ concentrations in the atmosphere are around 420 ppm [55]. CO_2_ readings when the bins were empty were in the range of 670–735 ppm, but there was still the occasional zero value, suggesting that the zero values were spurious values or glitches in the CO_2_ meters. There were far more zero or lower-than-limit-of-detection observations for the CO_2_ values in the clean corn than in the dirty corn. The average CO_2_ concentration, RH and temperatures for the non-zero values were calculated for each sensor, and the environmental conditions were compared between clean and dirty corn, different layer depths, edge and center locations, and temperature/RH treatments.

Prior to the experimental set-up, the average levels of four mycotoxins (aflatoxin, deoxynivalenol, fumonisin and zearalenone) in thoroughly mixed clean and dirty grain were measured, and the mycotoxin concentrations are shown in Table 2. Due to the thorough mixing of the clean and dirty corn prior to loading the bins, it is assumed that initial distributions of fungal spores or mycotoxins was random and largely homogeneous within each bin. This assumption of a homogeneous background distribution of fungi is based on the thorough mixing process, which aims to evenly distribute any pre-existing aflatoxin contamination of corn kernels in each bin. While this mixing process does not guarantee a specific initial distribution of mycotoxins, it helps to ensure that any observed differences in mycotoxin levels between locations within a bin after storage can be attributed to the varying environmental conditions rather than pre-existing heterogeneity in the fungal distribution. These limiting assumptions should be noted, as the only way to control for position would be to have measurements in each location at the start and end of the storage period. This, however, is not possible as the samples themselves are destroyed by determining mycotoxin levels, and taking samples from near each sensor requires complete disturbance of the grain pile and thus is only possible at the end of the experiment.

As this is an exploratory, preliminary study to determine the feasibility of implementing this research at larger spatial and temporal scales, the six bins were stored as pairs in just three sets of environmental conditions for two months:

(1) 21 °C and 30% RH;
(2) 27 °C and 50% RH;
(3) 32 °C and 90% RH.

The chosen temperature/RH combinations represent a limited set of conditions, which may affect the generalizability of the results. Future studies should explore a wider range of temperature and RH levels to provide a more comprehensive understanding of mycotoxin distribution under various storage conditions. Future experimental plans are to temporally replicate the bins stored in these conditions and also to explore three RH levels at each storage temperature.

Following the two-month storage period at a given temperature and RH, two samples (~100 g) were taken from each of the 12 sensor locations in the bins of clean and dirty corn. Taking two samples from each sensor location allowed for some replication of corn kept under the same environmental conditions and in the same spatial position while balancing the cost and feasibility of the study. The samples were placed in a sterile bag until ground. Corn samples were ground in a Black & Decker coffee grinder (CBG110S, Spectrum Brands, Middleton, WI, USA) and sifted through a 20-mesh sieve for subsequent mycotoxin analysis.

### 4.2. Mycotoxin Analysis

The QuickScan II and the TotalTox Comb for Bulk Grain Corn test kits (EnviroLogix, Portland, ME, USA) were used according to the manufacturers’ instructions. A previous study demonstrated that aflatoxins exhibited a linear dose–response from 0 to 100 ppb when evaluated with this assay and compared LOD, LOQ, relative recovery and relative bias calculations against data obtained through HPLC. Correlations between the HPLC values and the QuickScan results were robust with a linear regression R^2^ value of 0.9897 [56]. While the QuickScan II and TotalTox Comb test kits provide rapid and cost-effective mycotoxin analysis, they may have some limitations compared to validated HPLC methods, such as lower sensitivity or specificity. The validation against HPLC methods helps ensure the reliability of the test kit results. While HPLC was identified as a more sensitive means of measuring aflatoxins, these data distinguish the QuickScan from the TotalTox Comb as a viable means for measuring mycotoxins. Additionally, prior to evaluation, the accuracy of this system was further evaluated in a preliminary trial against a validated HPLC method [57] and compared with results obtained from the QuickScan II using aflatoxin, deoxynivalenol, fumonisin and zearalenone analytical standards. No significant differences were observed.

For sample evaluation, a 25 g sample of ground corn, passed through a 20-mesh sieve, was placed into a sterile jar with 75 mL distilled water. The extraction buffer pouch (EB17) provided by the kit provider was added, allowed to dissolve and shaken by hand for 2 min. The extract was centrifuged for 30 s at 2000× *g* and the top layer was removed. Then, 100 µL of DB5 buffer and 100 µL of the extracted solution were combined in sterile test tubes and acclimated for two min at room temperature (21–23 °C). Test combs were added to the tubes (with arrows pointing down) and reacted for 4 min. The test strips were then cut as instructed by the manufacturer before being placed into the calibrated QuickScan II for analysis. The system software provided quantitative results, with aflatoxin and zearalenone levels provided in ppb, and DON and fumonisin in ppm. For aflatoxin-contaminated corn, the samples were serially diluted as per the manufacturer’s instructions due to the high levels of toxin present.

### 4.3. Statistical Methods

In an ideal laboratory experiment, as many variables are kept constant as possible to be able to identify the effects of treatments. In environmental and spatial studies, however, it is impossible to control for all variables apart from one. Therefore, within the spatial sciences, where several variables change with location, the only option is to compare environmental conditions with changes in location or toxin concentrations with changes in environmental conditions. Environmental data collected from each of the sensors were examined. For each dataset, there were many readings, with a value of zero interspersed with non-zero values. Data with non-zero CO_2_ concentrations were investigated for each sensor, and the minimum, maximum and mean CO_2_ concentrations, temperatures and humidities for each sensor were calculated. The data were then collated for the clean and dirty bins for all three temperature and humidity combinations (*n* = 72) and just dirty bins (*n* = 36). Combining data from both bins and all temperature combinations gave larger datasets and allowed for the comparison of the environmental conditions and mycotoxin concentrations based on several different variables. The mean CO_2_ concentrations, humidities and temperatures from each sensor between clean and dirty bins and between center and edge locations were compared using the Mann–Whitney U test for both datasets (*n* = 72 and *n* = 36). The mean CO_2_ concentrations, humidities and temperatures from each sensor between layers in the bins and between different temperature/RH treatments were compared using the Kruskal–Wallis H test.

Data for the concentrations of each mycotoxin at the different sensor nodes (2 measurements per sensor) were compiled to create datasets which included a total dataset with all temperature/RH combinations and clean and dirty corn (*n* = 144, e.g., Table 3). Datasets including only dirty or clean corn were also collated (*n* = 72, e.g., Table 3) and datasets which only included information for the clean and dirty corn at one temperature were also compiled (*n* = 48, e.g., Table 3). The summary statistics of these datasets were calculated, and due to several of the datasets showing coefficients of skewness greater than 1 (Table 3), non-parametric comparison tests were used for further analysis. The Mann–Whitney U test was used for two-sample comparisons such as comparing mycotoxin concentrations between the clean and dirty corn and between central and edge locations. The Kruskal–Wallis H test was used for three-sample comparisons such as comparing mycotoxin concentrations between layers in the storage bins (top, middle, bottom) and between storage temperatures/humidities. All statistical analyses were completed in SPSS version 28 [58].

## Figures and Tables

**Table 1:** Optimal temperature and moisture conditions for fungal growth and mycotoxin production for aflatoxin, deoxynivalenol (DON), fumonisin and zearalenone.

Mycotoxin	Aflatoxin	DON	Fumonisin	Zearalenone
**FDA Legislative Limits (ppb) (Ref.)**	20 ppb, 100 ppb, 200 ppb, 300 ppb (FDA 2015)	1000 ppb FDA (2010)	4000 ppb FDA (2001)	No limit yet established by FDA
**Fungal Species Producing Toxin**	*Aspergillus flavus* *Aspergillus parasiticus*	*Fusarium graminearum*	*Fusarium verticillioides* *Fusarium proliferatum*	*Fusarium graminearum* *Fusarium culmorum*
**Optimal Temperature for Fungal Growth (°C)**	30 °C	25 °C	30 °C	25 °C
**Optimal ERH for** **Fungal Growth** **(%)**	96	99.5	96.9	99.5
**Optimal Temperature for Mycotoxin** **Production (°C)**	37 °C	20–30 °C	22 °C	28 °C
**Optimal ERH for** **Mycotoxin Production (%)**	99	97	97.2	97
**References**	Samapundo et al. (2007) [19]	Belizán et al. (2019) [16]	Samapundo et al. (2005) [18]	Jiménez et al. (1996) [17]

ERH is equilibrium relative humidity.

**Table 2:** Initial mycotoxin concentrations in clean and dirty corn based on averages of 12 random samples taken from thoroughly mixed corn.

	Aflatoxin (ppb)	DON (ppb)	Fumonisin (ppm)	Zearalenone (ppb)
clean	0	28	3.46	22
dirty	300	29	5.21	115

DON is deoxynivalenol. Clean and dirty refer to very low or high concentrations of aflatoxin present in the corn.

**Table 3:** Aflatoxin (ppb) dataset summary statistics.

Conditions	N	Minimum	Maximum	Mean	Std. Deviation	Skewness
21–32 °C, 30–90% RH clean and dirty	144	0	3200	598	805	1.29
21–32 °C, 30–90% RH dirty only	72	200	3200	1195	762	0.69
21–32 °C, 30–90% RH clean only	72	0	4.5	0.38	1.05	2.76
21 °C, 30% RH clean and dirty	48	0	2200	549	709	0.99
27 °C, 50% RH clean and dirty	48	0	2900	697	893	1.13
32 °C, 90% RH clean and dirty	48	0	3200	547	810	1.67

Clean and dirty refer to very low or high concentrations of aflatoxin present in the corn.

**Table 4:** Mean concentrations of mycotoxins between layers and between central and edge locations in the grain bins stored under different conditions.

	Clean						Dirty						21–32 °C, 30–90% RH						21 °C 30% RH						27 °C 50% RH						32 °C 90% RH					
	Bottom		Middle		Top		Bottom		Middle		Top		Bottom		Middle		Top		Bottom		Middle		Top		Bottom		Middle		Top		Bottom		Middle		Top	
Aflatoxin (ppb)	0.80		0.35		0.00		1817		1207		562		909		604		281		719		740		188		938		721		433		1069		349		222	
Zearalenone (ppb)	68		69		71		73		72		66		71		70		69		81		89		96		33		29		22		98		92		88	
	**Clean**						**Dirty**						**21–32 °C, 30–90% RH**						**21 °C 30% RH**						**27 °C 50% RH**						**32 °C 90% RH**					
	**Edge**			**Center**			**Edge**			**Center**			**Edge**			**Center**			**Edge**			**Center**			**Edge**			**Center**			**Edge**			**Center**		
Aflatoxin (ppb)	0.35			0.48			1256			1013			628			507			599			399			692			713			593			408		
Zearalenone (ppb)	68			74			72			64			70			69			84			104			30			21			96			83		

Clean and dirty refer to very low or high concentrations of aflatoxin present in the corn.

**Table 5:** Zearalenone (ppb) dataset summary statistics.

Conditions	N	Minimum	Maximum	Mean	Std. Deviation	Skewness
21–32 °C, 30–90% RH clean and dirty	144	0.12	250	70	38	0.55
21–32 °C, 30–90% RH dirty only	72	20	130	70	28	0.06
21–32 °C, 30–90% RH clean only	72	0.12	250	69	47	0.62
21 °C, 30% RH clean and dirty	48	45	250	89	34	2.82
27 °C, 50% RH clean and dirty	48	0.12	73	28	19	0.49
32 °C, 90% RH clean and dirty	48	62	130	93	16	−0.10

Clean and dirty refer to very low or high concentrations of aflatoxin present in the corn.

## Data Availability

The original contributions presented in the study are included in the article/Supplementary Material, further inquiries can be directed to the corresponding author/s.

## Supplementary Materials

The following supporting information can be downloaded at: https://www.mdpi.com/article/10.3390/toxins16120508/s1, Figure S1: 3D plots showing the spatial distribution of DON (ppb) build-up in clean and dirty corn stored at three temperature/RH combinations (21 °C 30% RH, 27 °C 50% RH and 32 °C 90% RH).; Figure S2: Box plots showing the comparison in DON levels (ppb) between different layers, center and edge locations and clean and dirty corn stored at different temperature/RH combinations (21 °C 30% RH, 27 °C 50% RH and 32 °C 90% RH).; Figure S3. Box plots showing Deoxynivalenol (DON) and Fumonisin levels between different layers, center and edge loca-tions and temperature/RH combinations (21 °C 30% RH, 27 °C 50% RH and 32 °C 90% RH) for (a) only clean and (b) dirty only stored corn Figure S4: 3D plots showing the spatial distribution of Fumonisin (ppm) build-up in “clean” and “dirty” corn stored at three temperatures/ RH combinations (21 °C 30% RH, 27 °C 50% RH and 32 °C 90% RH).; Figure S5: Box plots showing the comparison in Fumonisin levels (ppm) between different layers, cen-ter and edge locations and “clean” and “dirty” corn stored at different temperature/RH combinations (21 °C 30% RH, 27 °C 50% RH and 32 °C 90% RH).; Table S1. Summary of Comparison Tests (Mann Whitney U and Kruskal Wallis H Tests) for Aflatoxin, Table S2. Summary of Comparison Tests (Mann Whitney U and Kruskal Wallis H Tests) for Zearalenone; Table S3: DON (ppb) Datasets Summary Statistics; Table S4: Summary of Comparison Tests (Mann Whitney U and Kruskal Wallis H Tests) for DON; Table S5. Mean Concentrations of Mycotoxins Be-tween Layers and Between Center and Edge Locations in the Grain Bins Stored Under Different Conditions; Table S6: Fumonisin (ppm) Datasets Summary Statistics; Table S7: Summary of Comparison Tests (Mann Whitney U and Kruskal Wallis H Tests) for Fumonisin.
