# Supplementary material for: An Investigation of the Spatial Arrangement of Mycotoxin Build-Up in Corn Stored Under Different Environmental Conditions
Source: Toxins (Basel). 2024 Nov 25;16(12):508. doi: 10.3390/toxins16120508 (PMC11728487; doi:10.3390/toxins16120508)
Supplement: Supplementary file 1 [file toxins-16-00508-s001.zip › toxins-3268323-supplementary.pdf]

Supplementary Materials: An Investigation of the Spatial Arrangement of Mycotoxin Build-Up in Corn Stored Under Different Environmental Conditions

Ruth Kerry, Ben Ingram, Hamed K. Abbas and Gene Ahlborn

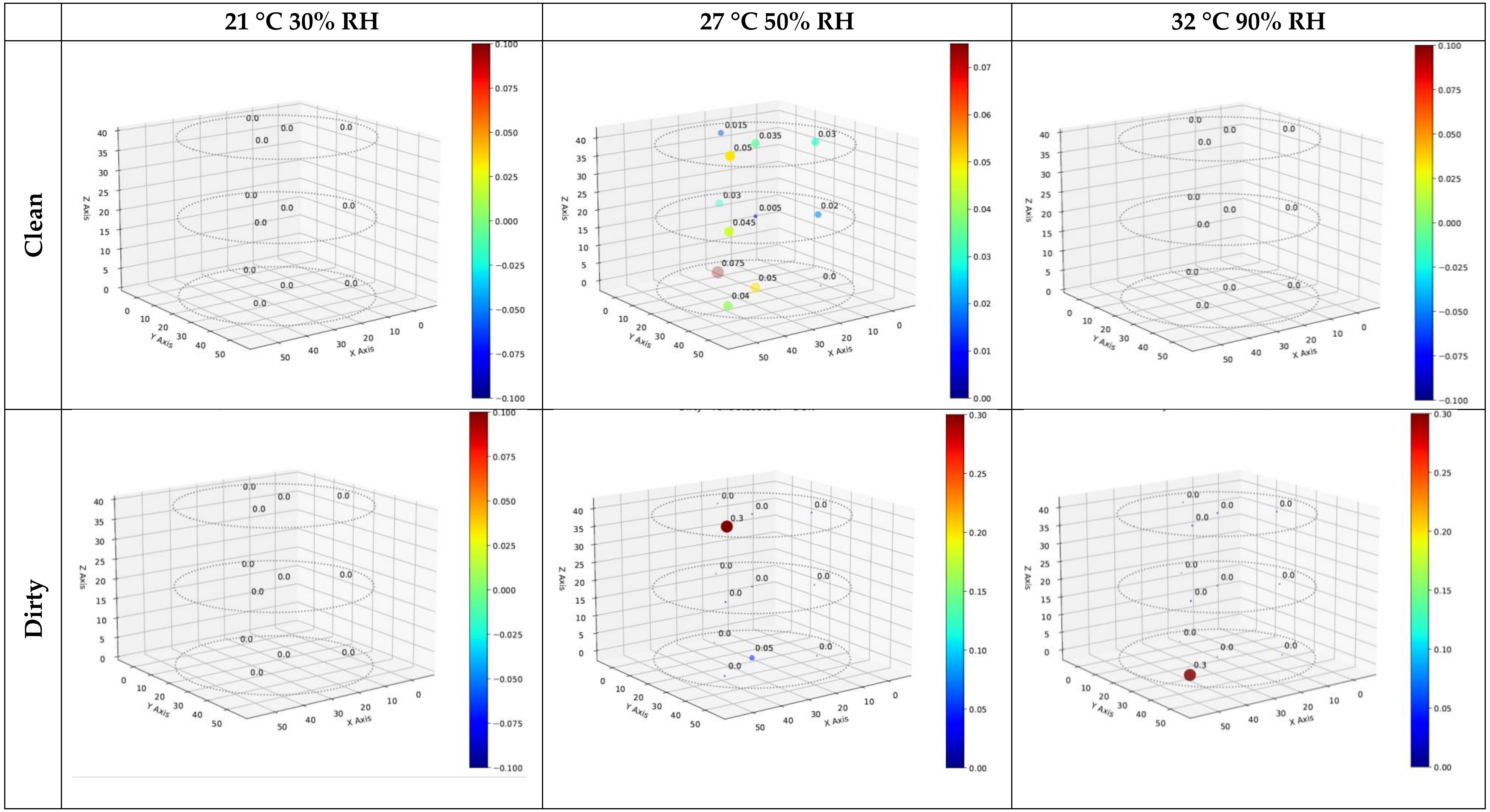

Figure S1. 3D plots showing the spatial distribution of DON (ppb) build-up in clean and dirty corn stored at three temperature/RH combinations (21 °C 30% RH, 27 °C 50% RH and 32 °C 90% RH).

Each colored dot shows the average toxin concentration of two samples.

	21 °C 30% RH	27 °C 50% RH	32 °C 90% RH	All Temperatures/RHs
**Layer Depth**	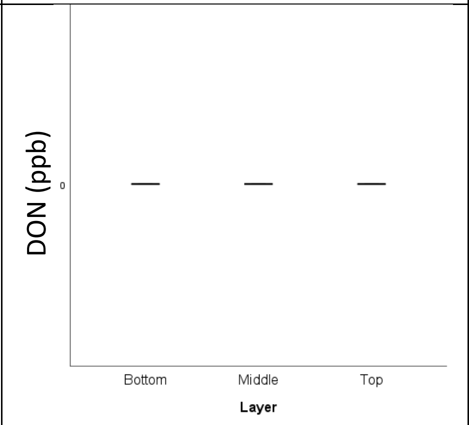 *p*=1.000	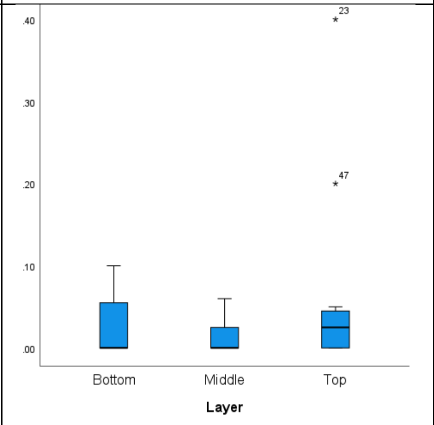 *p*=0.433	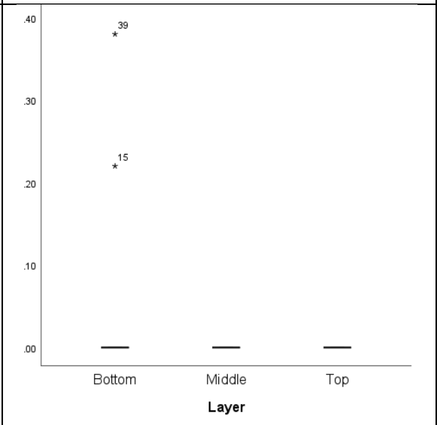 *p*=0.130	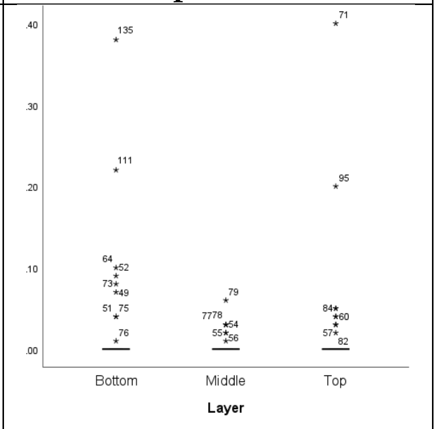 *p*=0.677
**Center vs edge**	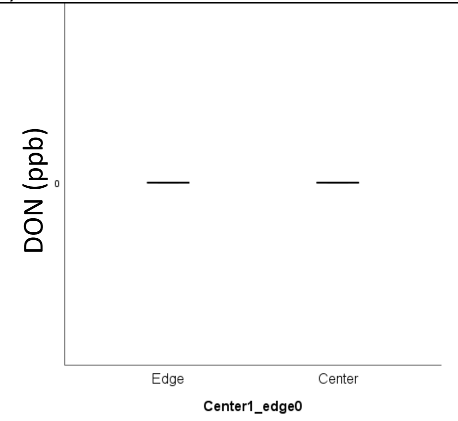 *p*=1.000	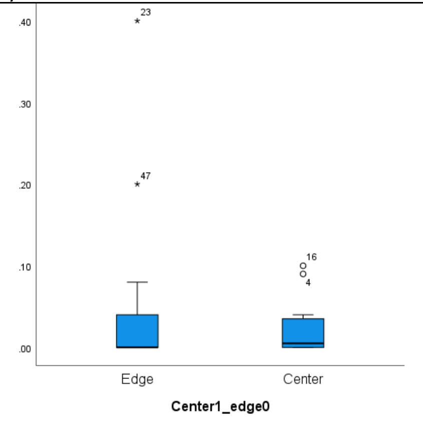 *p*=0.969	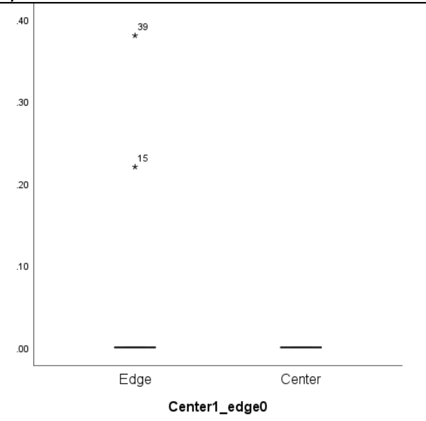 *p*=0.409	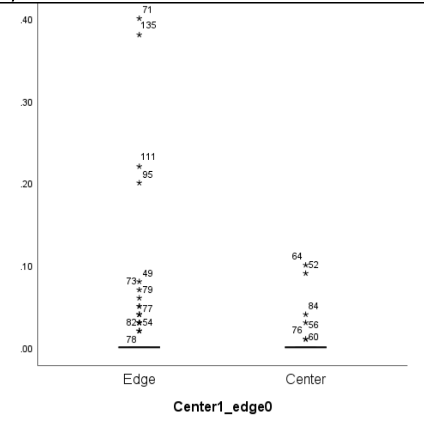 *p*=0.826
**Clean vs Dirty**	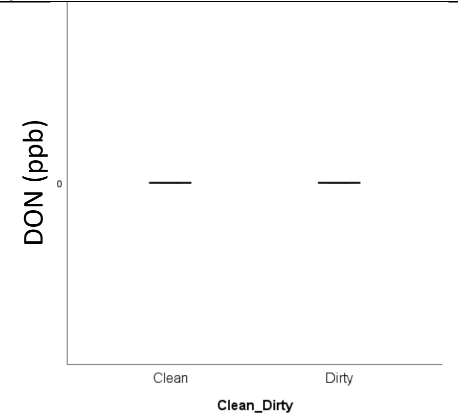 *p*=1.000	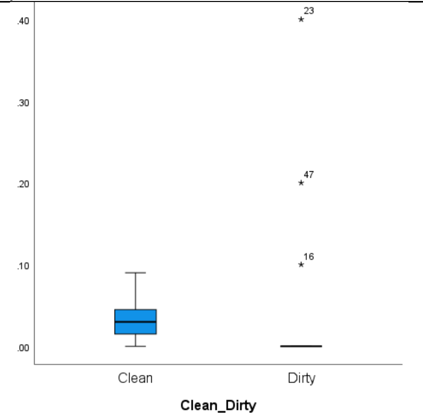 *p*<0.001	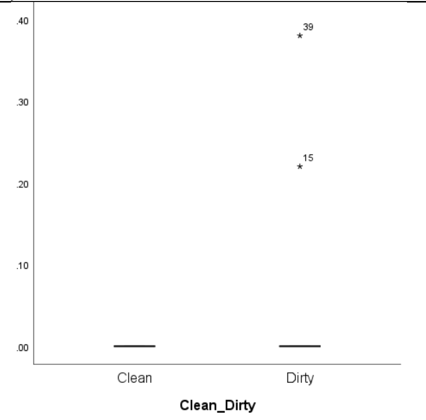 *p*=0.153	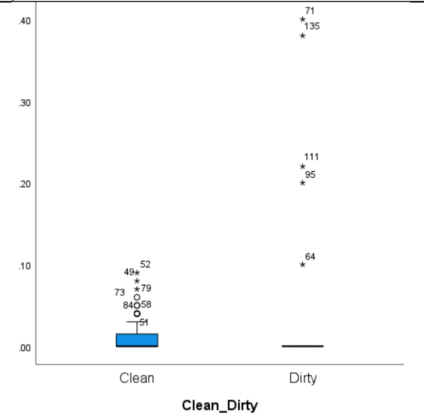 *p*=0.003

Figure S2. Box plots showing the comparison in DON levels (ppb) between different layers, center and edge locations and clean and dirty corn stored at different temperature/RH combinations (21 °C 30% RH, 27 °C 50% RH and 32 °C 90% RH).

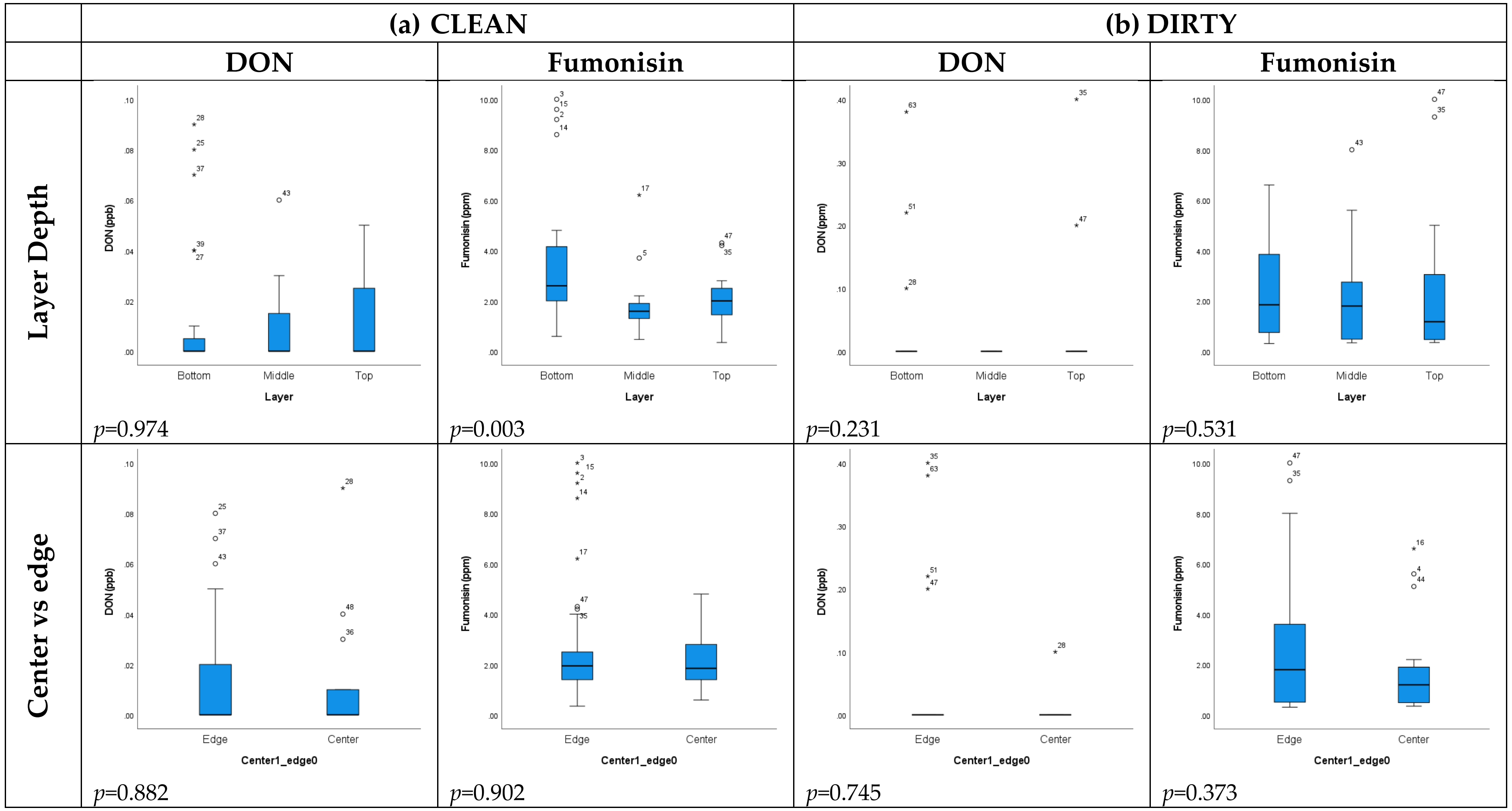

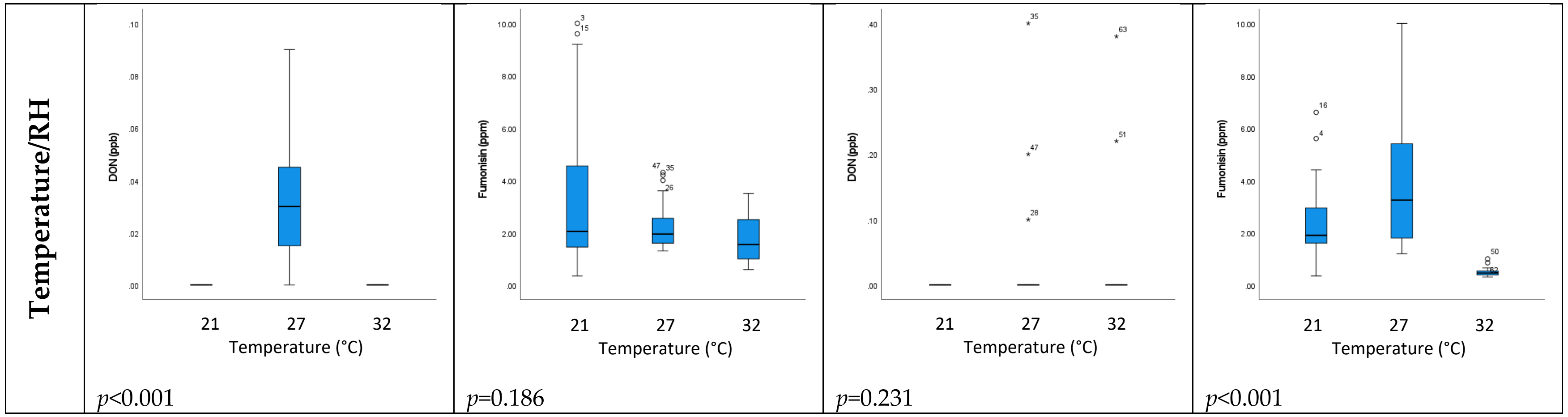

Figure S3. Box plots showing Deoxynivalenol (DON) and Fumonisin levels between different layers, center and edge locations and temperature/RH combinations (21 °C 30% RH, 27 °C 50% RH and 32 °C 90 % RH) for (a) only clean and (b) dirty only stored corn. *Clean and dirty refer to very low or high concentrations of aflatoxin present in the corn.*

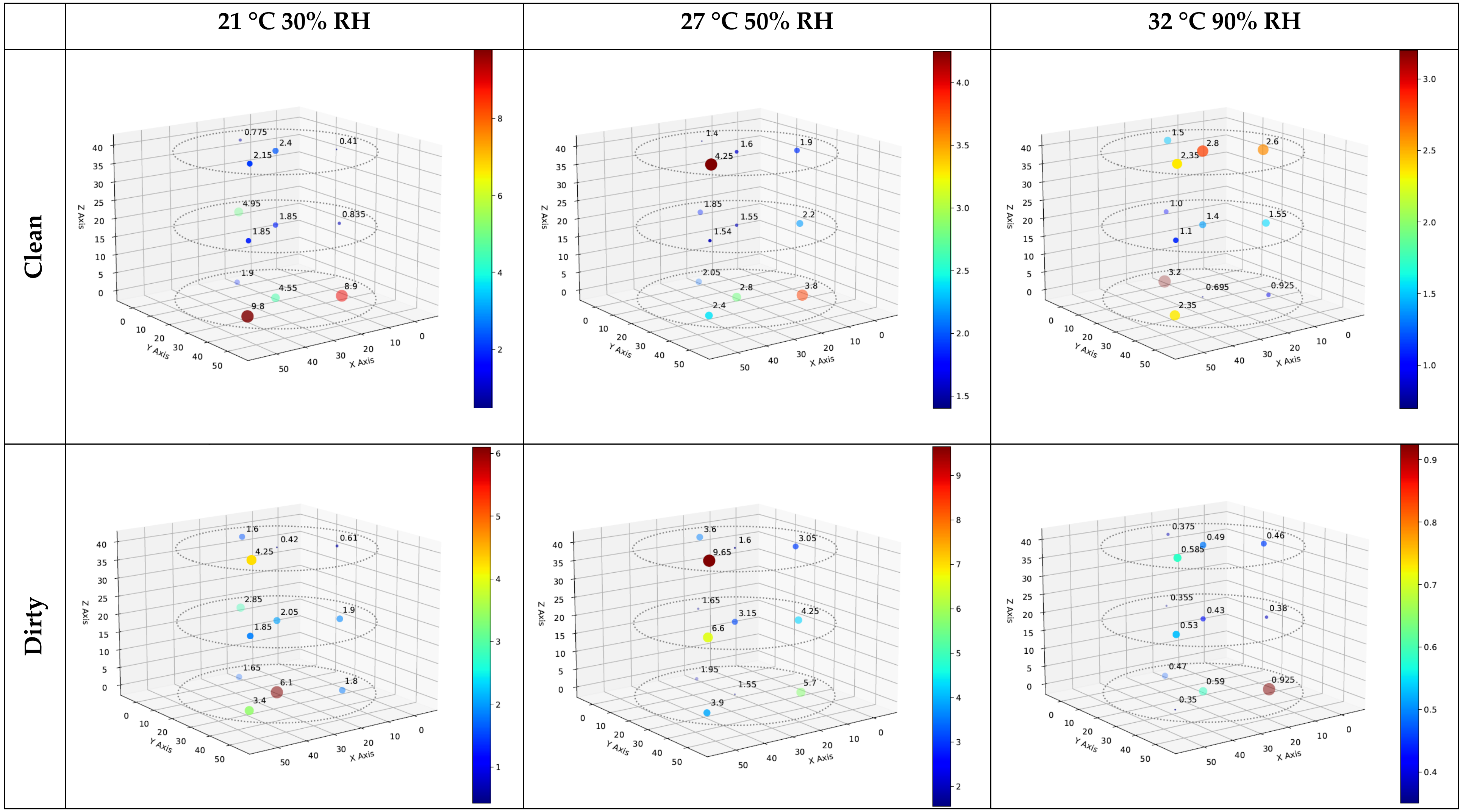

Figure S4. 3D plots showing the spatial distribution of Fumonisin (ppm) build-up in “clean” and “dirty” corn stored at three temperatures/ RH combinations (21 °C 30% RH, 27 °C 50% RH and 32 °C 90% RH).

Each colored dot shows the average toxin concentration of two samples.

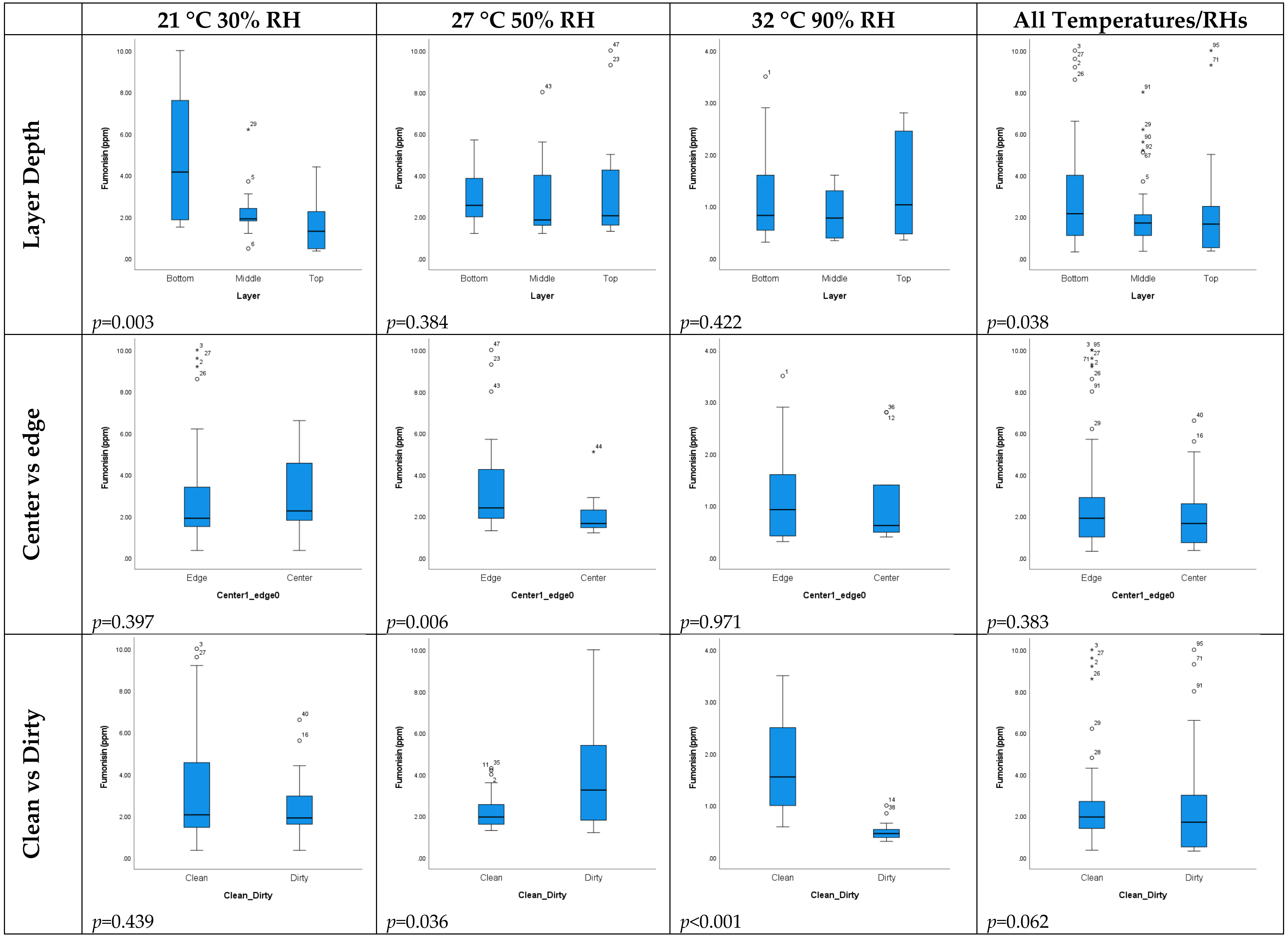
21 °C 30% RH
27 °C 50% RH
32 °C 90% RH
All Temperatures/RHs
Layer Depth
Center vs edge
Clean vs Dirty
Fumonisin (ppm)
Bottom
Middle
Top
Layer
Edge
Center
Center1_edge0
Clean
Dirty
Clean_Dirty
p=0.003
p=0.384
p=0.422
p=0.038
p=0.397
p=0.006
p=0.971
p=0.383
p=0.439
p=0.036
p<0.001
p=0.062

Figure S5. Box plots showing the comparison in Fumonisin levels (ppm) between different layers, center and edge locations and "clean" and "dirty" corn stored at different temperature/RH combinations (21 °C 30% RH, 27 °C 50% RH and 32 °C 90% RH).

Table S1. Summary of Comparison Tests (Mann Whitney U and Kruskal Wallis H Tests) for Aflatoxin

Test Variable	N	Grouping variable	Order of classes (Low to High)	*p*-value
Aflatoxin: all temps/RH, clean and dirty	144	Layer Depth	Top, Middle, Bottom	0.018
Aflatoxin: all temps/RH, clean and dirty	144	Center vs Edge	Center, Edge	0.868
Aflatoxin: all temps/RH, clean and dirty	144	Clean vs Dirty	Clean, Dirty	<0.001
Aflatoxin: all temps/RH, clean and dirty	144	Temperature	32 °C, 21 °C, 27 °C	0.429
Aflatoxin: all temps/RH, clean only	72	Layer Depth	Top, Middle, Bottom	0.035
Aflatoxin: all temps/RH, clean only	72	Center vs Edge	Edge, Center	0.132
Aflatoxin: all temps/RH, clean only	72	Temperature	21 °C, 32 °C, 27 °C	0.011
Aflatoxin: all temps/RH, dirty only	72	Layer Depth	Top, Middle, Bottom	<0.001
Aflatoxin: all temps/RH, dirty only	72	Center vs Edge	Center, Edge	0.164
Aflatoxin: all temps/RH, dirty only	72	Temperature	32 °C, 21 °C, 27 °C	0.240
Aflatoxin: 21 °C 30% RH, clean and dirty	48	Layer Depth	Top, Bottom, Middle	0.280
Aflatoxin: 21 °C 30% RH, clean and dirty	48	Center vs Edge	Center, Edge	0.752
Aflatoxin: 21 °C 30% RH, clean and dirty	48	Clean vs Dirty	Clean, Dirty	<0.001
Aflatoxin: 27 °C 50% RH, clean and dirty	48	Layer Depth	Top, Middle, Bottom	0.204
Aflatoxin: 27 °C 50% RH, clean and dirty	48	Center vs Edge	Center, Edge	0.856
Aflatoxin: 27 °C 50% RH, clean and dirty	48	Clean vs Dirty	Clean, Dirty	<0.001
Aflatoxin: 32 °C 90% RH, clean and dirty	48	Layer Depth	Top, Middle, Bottom	0.171
Aflatoxin: 32 °C 90% RH, clean and dirty	48	Center vs Edge	Center, Edge	0.980
Aflatoxin: 32 °C 90% RH, clean and dirty	48	Clean vs Dirty	Clean, Dirty	<0.001

Clean and dirty refer to very low or high concentrations of aflatoxin present in the corn

Table S2. Summary of Comparison Tests (Mann Whitney U and Kruskal Wallis H Tests) for Zearalenone

Test Variable	N	Grouping variable	Order of classes (Low to High)	p-value
Zearalenone: all temps/RH, clean and dirty	144	Layer Depth	Top, Middle, Bottom	0.605
Zearalenone: all temps/RH, clean and dirty	144	Center vs Edge	Center, Edge	0.283
Zearalenone: all temps/RH, clean and dirty	144	Clean vs Dirty	Dirty, Clean	0.992
Zearalenone: all temps/RH, clean and dirty	144	Temperature	27 °C, 21 °C, 32 °C	<0.001
Zearalenone: all temps/RH, clean only	72	Layer Depth	Top, Bottom, Middle	0.487
Zearalenone: all temps/RH, clean only	72	Center vs Edge	Center, Edge	0.602
Zearalenone: all temps/RH, clean only	72	Temperature	27 °C, 32 °C, 21 °C	<0.001
Zearalenone: all temps/RH, dirty only	72	Layer Depth	Top, Bottom, Middle	0.833
Zearalenone: all temps/RH, dirty only	72	Center vs Edge	Center, Edge	0.304
Zearalenone: all temps/RH, dirty only	72	Temperature	27 °C, 21 °C, 32 °C	<0.001
Zearalenone: 21 °C 30% RH, clean and dirty	48	Layer Depth	Bottom, Top, Middle	0.713
Zearalenone: 21 °C 30% RH, clean and dirty	48	Center vs Edge	Edge, Center	0.712
Zearalenone: 21 °C 30% RH, clean and dirty	48	Clean vs Dirty	Dirty, Clean	<0.001
Zearalenone: 27 °C 50% RH, clean and dirty	48	Layer Depth	Top, Middle, Bottom	0.315
Zearalenone: 27 °C 50% RH, clean and dirty	48	Center vs Edge	Center, Edge	0.127
Zearalenone: 27 °C 50% RH, clean and dirty	48	Clean vs Dirty	Clean, Dirty	<0.001
Zearalenone: 32 °C 90% RH, clean and dirty	48	Layer Depth	Top, Middle, Bottom	0.305
Zearalenone: 32 °C 90% RH, clean and dirty	48	Center vs Edge	Center, Edge	0.005
Zearalenone: 32 °C 90% RH, clean and dirty	48	Clean vs Dirty	Clean, Dirty	<0.001

Clean and dirty refer to very low or high concentrations of aflatoxin present in the corn.

Table S3. DON (ppb) Datasets Summary Statistics

Conditions	N	Minimum	Maximum	Mean	Std. Deviation	Skewness
21-32 °C, 30-90% RH clean and dirty	144	0	0.40	0.01	0.05	5.61
21-32 °C, 30-90% RH dirty only	72	0	0.40	0.02	0.07	4.36
21-32 °C, 30-90% RH clean only	72	0	0.09	0.01	0.02	2.05
21 °C, 30% RH clean and dirty	48	0	0.00	0.00	0.00	0
27 °C, 50% RH clean and dirty	48	0	0.40	0.03	0.07	4.27
32 °C, 90% RH clean and dirty	48	0	0.38	0.01	0.06	5.25

Table S4. Summary of Comparison Tests (Mann Whitney U and Kruskal Wallis H Tests) for DON

Test Variable	N	Grouping variable	Order of classes (Low to High)	*p*-value
DON: all temps/RH, clean and dirty	144	Layer Depth	Middle, Top, Bottom	0.677
DON: all temps/RH, clean and dirty	144	Center vs Edge	Center, Edge	0.826
DON: all temps/RH, clean and dirty	144	Clean vs Dirty	Dirty, Clean	0.003
DON: all temps/RH, clean and dirty	144	Temperature	21 °C, 32 °C, 27 °C	<0.001
DON: all temps/RH, clean only	72	Layer Depth	Middle, Bottom, Top	0.974
DON: all temps/RH, clean only	72	Center vs Edge	Center, Edge	0.882
DON: all temps/RH, clean only	72	Temperature	21 °C, 32 °C, 27 °C	<0.001
DON: all temps/RH, dirty only	72	Layer Depth	Middle, Top, Bottom	0.231
DON: all temps/RH, dirty only	72	Center vs Edge	Center, Edge	0.745
DON: all temps/RH, dirty only	72	Temperature	21 °C, 32 °C, 27 °C	0.231
DON: 21 °C 30% RH, clean and dirty	48	Layer Depth	Equal	1.000
DON: 21 °C 30% RH, clean and dirty	48	Center vs Edge	Equal	1.000
DON: 21 °C 30% RH, clean and dirty	48	Clean vs Dirty	Equal	1.000
DON: 27 °C 50% RH, clean and dirty	48	Layer Depth	Middle, Bottom, Top	0.433
DON: 27 °C 50% RH, clean and dirty	48	Center vs Edge	Center, Edge	0.969
DON: 27 °C 50% RH, clean and dirty	48	Clean vs Dirty	Dirty, Clean	<0.001

DON: 32 °C 90% RH, clean and dirty	48	Layer Depth	Top-Middle, Bottom	0.130
DON: 32 °C 90% RH, clean and dirty	48	Center vs Edge	Center, Edge	0.409
DON: 32 °C 90% RH, clean and dirty	48	Clean vs Dirty	Clean, Dirty	0.153

Table S5. Mean Concentrations of Mycotoxins Between Layers and Between Center and Edge Locations in the Grain Bins Stored Under Different Conditions

	**Clean**			**Dirty**			**21-32 °C, 30-90% RH**			**21 °C 30% RH**			27 °C 50% RH			32 °C 90% RH		
	**Bottom**	**Middle**	**Top**	**Bottom**	**Middle**	**Top**	**Bottom**	**Middle**	**Top**	**Bottom**	**Middle**	Top	Bottom	Middle	Top	Bottom	Middle	Top
DON (ppm)	0.01	0.01	0.01	0.03	0.00	0.03	0.02	0.00	0.02	0.00	0.00	0.00	0.03	0.01	0.05	0.04	0.00	0.00
Fumonisin (ppm)	3.61	1.81	2.01	2.37	2.17	2.22	2.99	1.99	2.12	4.76	2.27	1.58	3.02	2.85	3.38	1.19	0.84	1.40

	Clean		Dirty		21-32 °C, 30-90% RH		21 °C 30% RH		27 °C 50% RH		32 °C 90% RH	
	Edge	Centre	Edge	Centre	Edge	Centre	Edge	Centre	Edge	Centre	Edge	Centre
DON (ppm)	0.01	0.01	0.02	0.01	0.02	0.01	0.00	0.00	0.03	0.02	0.02	0.00
Fumonisin (ppm)	2.58	2.18	2.40	1.82	2.49	2.00	2.86	2.90	3.43	2.04	1.17	1.07

Clean and dirty refer to very low or high concentrations of aflatoxin present in the corn DON is deoxynivalenol

Table S6. Fumonisin (ppm) Datasets Summary Statistics

**Conditions**	**N**	**Minimum**	**Maximum**	**Mean**	**Std. Deviation**	**Skewness**
21-32 °C, 30-90% RH clean and dirty	144	0.31	10	2.36	2.10	1.91
21-32 °C, 30-90% RH dirty only	72	0.31	10	2.25	2.21	1.60
21-32 °C, 30-90% RH clean only	72	0.35	10	2.48	1.99	2.41
21 °C, 30% RH clean and dirty	48	0.35	10	2.87	2.47	1.61
27 °C, 50% RH clean and dirty	48	1.20	10	3.08	2.06	1.77
32 °C, 90% RH clean and dirty	48	0.31	3.5	1.14	0.88	1.10

Table S7. Summary of Comparison Tests (Mann Whitney U and Kruskal Wallis H Tests) for Fumonisin

Test Variable	N	Grouping variable	Order of classes (Low to High)	p-value
Fumonisin: all temps/RH, clean and dirty	144	Layer Depth	Middle, Top, Bottom	0.038
Fumonisin: all temps/RH, clean and dirty	144	Center vs Edge	Center, Edge	0.383
Fumonisin: all temps/RH, clean and dirty	144	Clean vs Dirty	Dirty, Clean	0.062
Fumonisin: all temps/RH, clean and dirty	144	Temperature	32 °C, 21 °C, 27 °C	<0.001
Fumonisin: all temps/RH, clean only	72	Layer Depth	Middle, Top, Bottom	0.003
Fumonisin: all temps/RH, clean only	72	Center vs Edge	Edge, Center	0.902
Fumonisin: all temps/RH, clean only	72	Temperature	32 °C, 27 °C, 21 °C	0.186
Fumonisin: all temps/RH, dirty only	72	Layer Depth	Top, Middle, Bottom	0.531
Fumonisin: all temps/RH, dirty only	72	Center vs Edge	Center, Edge	0.373
Fumonisin: all temps/RH, dirty only	72	Temperature	32 °C, 21 °C, 27 °C	<0.001
Fumonisin: 21 °C 30% RH, clean and dirty	48	Layer Depth	Top, Middle, Bottom	0.003
Fumonisin: 21 °C 30% RH, clean and dirty	48	Center vs Edge	Edge, Center	0.397
Fumonisin: 21 °C 30% RH clean and dirty	48	Clean vs Dirty	Dirty, Clean	0.439
Fumonisin: 27 °C 50% RH, clean and dirty	48	Layer Depth	Middle, Top, Bottom	0.384
Fumonisin: 27 °C 50% RH, clean and dirty	48	Center vs Edge	Center, Edge	0.006
Fumonisin: 27 °C 50% RH, clean and dirty	48	Clean vs Dirty	Clean, Dirty	0.036
Fumonisin: 32 °C 90% RH, clean and dirty	48	Layer Depth	Middle, Bottom, Top	0.422
Fumonisin: 32 °C 90% RH clean and dirty	48	Center vs Edge	Center, Edge	0.971
Fumonisin: 32 °C 90% RH, clean and dirty	48	Clean vs Dirty	Dirty, Clean	<0.001
